# From Exposure to Atherosclerosis: Mechanistic Insights into Phthalate-Driven Ischemic Heart Disease and Prevention Strategies

**DOI:** 10.3390/life16020327

**Published:** 2026-02-13

**Authors:** Francesca Gorini, Alessandro Tonacci, Mariangela Palazzo, Andrea Borghini

**Affiliations:** Institute of Clinical Physiology, National Research Council, 56124 Pisa, Italy; alessandro.tonacci@cnr.it (A.T.); mariangelapalazzo@cnr.it (M.P.)

**Keywords:** phthalates, ischemic heart disease, atherosclerosis, oxidative stress, inflammation, mitochondrial dysfunction, DNA methylation, non-coding RNA, lipid accumulation, artificial intelligence

## Abstract

Despite decades of interventions targeting modifiable risk factors to reduce the burden of cardiovascular disease, ischemic heart disease (IHD) remains the leading cause of mortality and the second leading cause of disability-adjusted life-years worldwide. Growing evidence suggests that phthalates–plasticizers widely used in consumer products, cosmetics, and medical devices, and therefore ubiquitous across environmental media, may contribute to IHD development. Epidemiological studies have reported associations between phthalate exposure and multiple markers of atherosclerosis, the pathological hallmark of IHD, with or without mediation by traditional cardiovascular risk factors. Experimental models support these findings, showing that phthalates can induce oxidative stress, mitochondrial dysfunction, apoptosis, lipid accumulation, and epigenetic alterations, all of which promote endothelial damage and atherogenesis. In this review, we synthesize current epidemiological findings linking phthalate exposure to IHD, describe the main cellular and molecular mechanisms involved, and outline research gaps and regulatory perspectives. We also discuss how novel analytical frameworks—including artificial intelligence—may enhance the integration of environmental, clinical, and molecular data to advance risk prediction and prevention strategies.

## 1. Introduction

Despite a progressive decline in the public health burden of cardiovascular disease (CVD) over the past few decades—largely due to interventions targeting modifiable risk factors such as tobacco control, and blood pressure and cholesterol-lowering strategies [1,2]—CVD remains a major global cause of mortality, with an estimated 19.8 million deaths in 2022, representing approximately 32% of all global deaths [3,4]. Conversely, the rising prevalence of obesity and diabetes, compounded by the recent impact of the COVID-19 pandemic, may have substantially and adversely influenced CVD trends [2]. While age-standardized CVD mortality and incidence rates decreased by 16.8% and 46.5% globally from 1990 to 2019, the global absolute number of incident cases and CVD deaths increased, respectively, by 77.1% and 53.8% in the same period, especially in low, low-middle, and middle sociodemographic index (SDI) regions [5]. According to the Global Burden of Disease Study 2021, ischemic heart disease (IHD) ranked as the second leading cause of disability-adjusted life-years globally and was the foremost cause of mortality, accounting for nearly 9 million deaths worldwide in that year [4,6]. Notably, the age-standardized rate of mortality due to IHD was lowest in the high SDI regions—51.0 per 100,000 population (95% Confidence Interval [CI]: 44.9–54.2)—and highest in the low-middle SDI quintile, particularly in North Africa and the Middle East, where it reached 202.8 per 100,000 population, highlighting how the global burden of IHD is exacerbated by sociodemographic disadvantages, including limited access to effective healthcare systems and inadequate implementation of primary prevention strategies [3,6,7].

IHD arises from the interplay between structural and functional alterations in the coronary arteries and the myocardium [8]. Together with ischemic stroke and peripheral arterial disease, IHD is classified under atherosclerotic cardiovascular diseases (ASCVDs), which share a common pathophysiological hallmark: atherosclerosis [9,10]. Atherosclerosis, characterized by localized thickening of artery walls due to intimal deposits of low-density lipoprotein, immune and vascular cell types, and extracellular matrix proteins, may lead to the development of atherosclerotic plaques in the aorta and its major distributing branches, including the coronary arteries [8,9]. Atherosclerotic plaques are central for clinical manifestations of ASCVD, ranging from stable ischemia such as chronic coronary syndrome, which may occur when plaques restrict blood flow, to more acute events resulting from plaque rupture or occlusion, including angina pectoris, transient ischemic attacks, and acute myocardial infarction (AMI) [9,10].

Both genetic and lifestyle determinants contribute to modulating the risk of IHD [11]. Individuals in the highest quintile of polygenic risk scores were shown to have a 91% increased risk of incident coronary events compared to those in the lowest quintile [11]. Importantly, adherence to a favorable lifestyle—defined as meeting at least three of the following criteria: absence of obesity, no current smoking, regular physical activity, and a healthy diet—was associated with a 46% lower relative risk of coronary events, even among individuals at high genetic risk [11]. Consistently, in addition to age, which is associated with a sharp increase in IHD risk, particularly in individuals aged 70 and older [7], a wide array of risk factors accelerate the development of atherosclerosis and, consequently, the risk of IHD [2], including hypertension [12], hypercholesterolemia [13], high fasting plasma glucose (FPG) [14], high body mass index (BMI) [15], smoking [16], dietary factors (e.g., processed and high-sodium foods typical of Westernized dietary patterns, inadequate intake of fruits and vegetables occurring in low SDI world regions) [17] and environmental pollution (e.g., air pollutants, toxic metals) [18,19,20].

The World Health Organization estimated that, in 2019, exposure to selected chemicals accounted for 2 million deaths and 53 million DALYs, nearly half of which were attributable to CVD [21]. Phthalic acid esters, or phthalates, are used as plasticizers in a wide range of consumer and industrial products including children’s toys, cosmetics, construction materials, food contact materials, and medical devices to enhance material flexibility and durability [22,23]. Global production of phthalates increased from approximately 2.7 to 6 million tons between 2007 and 2017, and it is currently estimated at approximately 11 million tons, with continued growth driven by low production costs and the absence of affordable alternatives [24,25,26]. As phthalates are not covalently bound to the polymer matrix, they can be gradually released into environmental media (soil, water, and air) through abrasion and leaching, and, owing to their semi-volatile nature, also via evaporation [26]. Phthalates are among the 1400 chemicals classified as potential endocrine disruptors, capable of significantly interfering with the endocrine system of animals and humans by mimicking or blocking the receptors of endogenous hormones [27,28]. In addition to associations with reproductive and neurodevelopmental health outcomes, as well as adverse birth outcomes (including low birth weight and preterm birth), emerging evidence suggests a possible role of phthalates in the development of obesity, type 2 diabetes (T2D), hypertension, atherosclerosis, and CVD [29,30,31]. Phthalate-induced cardiotoxicity may involve oxidative stress, hormonal effects, cardiomyocyte apoptosis, mitochondrial dysfunction, lipid accumulation, as well as inflammation-related mechanisms [30,32,33,34,35]. This review critically evaluates the current epidemiological evidence linking phthalate exposure to IHD, explores the underlying biological mechanisms, and identifies research gaps and future directions aimed at strengthening preventive strategies against IHD development.

## 2. Phthalates: An Overview

Phthalates are chemically defined as esters of 1,2-benzenedicarboxylic acid, formed through the reaction of alcohols with phthalic anhydride [36]. At room temperature, they are colorless and odorless compounds characterized by low melting points and high boiling points (>250 °C), properties largely influenced by the length of their alkyl chains [36]. Based on molecular weight, phthalates can be categorized into low-molecular-weight (LMW) phthalates, including butyl benzyl phthalate (BBP), di-n-butyl phthalate (DBP), diethyl phthalate (DEP), diisobutyl phthalate (DIBP), and dimethyl phthalate (DMP) and high-molecular-weight (HMW) phthalates, comprising 2-ethylhexyl phthalate (DEHP), diisononyl phthalate (DINP), and dinoctyl phthalate (DOP), among which BBP, DBP, DEHP, and DINP are the most widely used [37]. Phthalates are primarily added to enhance the durability and flexibility of plastics and vinyl and to prolong the persistence of fragrances [37]. Consequently, phthalates are present in a wide range of products, including coatings, vinyl flooring, food packaging, food processing materials, toys (typically containing HMW phthalates), as well as paints, adhesives, medical devises, cosmetics, shampoos, and perfumes (commonly containing LMW phthalates) [38].

**Figure 1:**
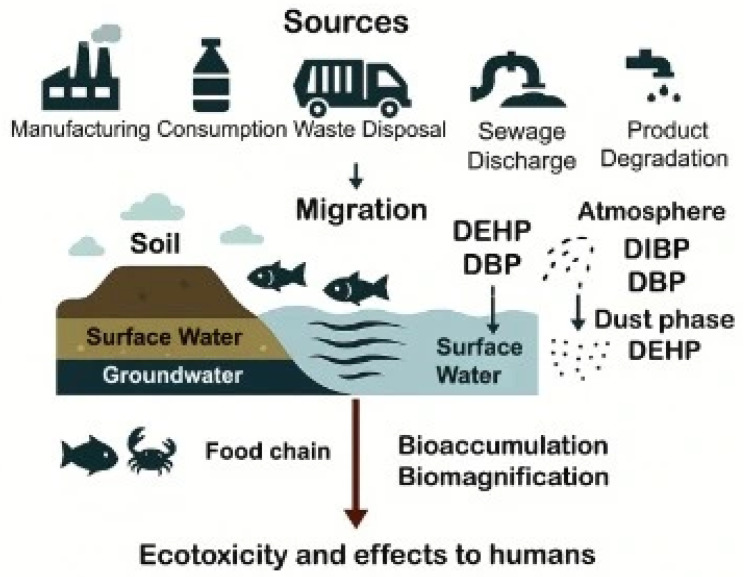
Sources and fate of phthalates in the environmental media. Image generated with AI Microsoft Copilot 365. Abbreviations: DBP: di-n-butyl phthalate; DEHP: 2-ethylhexyl phthalate; DIBP: 2-ethylhexyl phthalate.

Phthalates can accumulate in the environment as a result of manufacturing, consumption, improper waste segregation and disposal, sewage discharge, and, under certain conditions such as temperature, pH, contact time and type of interaction, the degradation of phthalate-containing products [38,39]. Owing to their semi-volatile nature, non-covalent association with polymers, and pronounced hydrophobicity, phthalates can readily migrate from their original matrix into soil, surface water, and groundwater, thereby posing a significant risk to both ecosystems and human health [38,39]. DEHP and DBP are the predominant phthalate species in aquatic systems, sediments, and soil, and they tend to bioaccumulate in aquatic invertebrates and fish and subsequently biomagnify along the food chain [40]. In the atmosphere, DIBP and DBP are the most representative species in the gas phase, whereas DEHP is predominant in the dust phase [40] (Figure 1). Importantly, the ecotoxicity of LMW phthalates appears to be higher than that of HMW species, largely due to their greater bioaccumulation potential [40].

Human exposure primarily occurs through food (up to 74%, [41])—particularly items high in animal fat, highly processed, or canned products—as well as drinking water stored in plastic and microwavable containers, dermal absorption of household and personal care products and, to a lesser extent, inhalation of contaminated vapors and dust [36,38]. Dietary exposure and food packaging films often contain HMW compounds (e.g., DEHP), along with elevated levels of DBP and DEP [42]. Daily dietary intake in adults is estimated to contribute between 1 and 4.2 µg/kg body weight (bw)/day of DEHP [43]. One relevant pathway of exposure to phthalates is the consumption of drinking water and other beverages stored in plastic bottles or containers [44] Although daily intake from bottled beverages generally remains below established toxicological thresholds, mean concentrations of total phthalates detected in carbonated water (7.43 μg/L) can exceed the U.S. Environmental Protection Agency’s reference level of 6 μg/L, with DEHP representing the major contributor [45,46]. Notably, long-term storage (180–365 days) increases phthalate migration into PET-bottled beverages [46]. In particular, DEP—a short-chain phthalate—tends to leach rapidly during the early storage period but subsequently undergoes degradation, whereas long-chain phthalates such as DEHP continue to accumulate over time [45,47]. If common storage conditions such as those found in retail outlets and supermarkets are generally considered safe for consumers, as concentrations of DBP, BBP, and DEHP typically remain low, factors including acidic pH, elevated temperatures, and prolonged light exposure can markedly accelerate phthalate migration [45,47].

DEHP has also been detected in house dust at median concentrations of approximately 700 mg/kg, with levels exceeding 3400 mg/kg in some samples [48,49]. Infants and toddlers are particularly vulnerable, as they incidentally ingest small amounts of dust and soil and frequently mouth plastic objects for several hours per day. Infants and toddlers are known to incidentally ingest small amounts of dust and soil, as well as mouth plastic objects for up to six hours per day [49]. This hand-to-mouth behavior can result in maximum exposures to DEHP and DINP exceeding 100 µg/kg bw/day—substantially higher than the tolerable daily intake (TDI) established for DEHP [36,49]. Additional exposure to phthalates may occur via medical devices made of polyvinyl chloride during procedures such as intravenous drug or total parenteral infusion, enteral feeding, cardiopulmonary bypass, hemodialysis, and blood transfusion [39]. Notably, DEHP released from medical devices can surpass the TDI, and may reach up to 21% of the device’s total DEHP content [39]. Once absorbed, phthalates undergo hydrolysis and oxidation, followed by conjugation, producing metabolites that are primarily excreted in urine [39]. These metabolites can be detected for up to 48 h, with half-lives ranging from 4 to 24 h, depending on the specific phthalate—HMW compounds exhibiting lower elimination—and the metabolite measured [39,50].

### Phthalate Regulations

The extensive use of phthalates, together with growing concerns about their harmful effects on human health, has prompted many countries to implement regulatory measures [51]. The European Union (EU), the world’s second-largest economy, was the first to introduce temporary restrictions on several phthalates—BBP, DEHP, DINP, di-iso-decyl phthalate (DIDP), and di-n-octyl phthalate (DnOP)—in children’s toys more than 25 years ago [52] (Table 1). The European Chemical Agency, in particular, coordinates the Registration, Evaluation, Authorization and Restriction of Chemicals (REACH), the EU’s primary legislative framework for protecting human health and the environment from chemical risks [53,54]. In 2006, Directive 2005/84/EC made permanent the restriction of DEHP, DnBP, and butylbenzyl phthalate (BBzP) in all toys and childcare articles (Annex XVII to REACH regulation No 1907/2006) [55]. Furthermore, DBP, DIBP, BBP, and DEHP—classified in the EU as Substances of Very High Concern and included in the Annex XIV authorization list due to their well-documented reproductive toxicity and endocrine-disrupting properties—have been restricted since 2020 to a maximum concentration of 0.1% by weight in plasticized material in all plastic products [53,56]. In 2019, the European Food Safety Authority (EFSA) Panel on Food Contact Materials, Enzymes, and Processing Aids issued an updated assessment establishing a TDI of 0.05 mg/kg body weight per day, expressed as DEHP equivalents, for DBP, BBP, DEHP, and DINP, based on their shared reproductive effects (i.e., reduction of fetal testosterone) [57]. For DIDP, the Panel maintained its individual TDI of 0.15 mg/kg body weight per day, based on liver toxicity [56]. EFSA also concluded that dietary exposure to these phthalates is four to seven times lower than the TDI for DBP, BBP, DEHP, and DINP, and approximately 1500 times lower than the TDI for DIDP [57]. EU Regulation No. 2021/2045 further expanded the scope of uses prohibited under entry No. 4 of REACH Annex XIV, setting 27 May 2025 as the sunset date for DEHP use in medical devices [58]. More recently, Regulation (EU) 2023/2482 extended the permitted use of DEHP in medical devices until 1 July 2030, provided that applications for authorization are submitted before 1 January 2029, thereby ensuring continued availability of essential medical devices during the transition to DEHP-free alternatives [59].

In December 2019, the United States Environmental Protection Agency (US EPA) classified BBP, DBP, DEHP, DIBP, and dicyclohexyl phthalate (DCHP) as High-Priority Substances, initiating formal risk evaluations [60]. Last year, the US EPA released the final risk evaluation for DIDP, identifying an unreasonable risk of injury to the health of female workers of reproductive age, but no risk for consumers, the general population, or the environment [61]. In contrast, the final risk evaluation for DINP concluded that the compound poses an unreasonable risk to human health, including developmental toxicity, hepatotoxicity, and cancer at higher exposure levels [62] (Table 1).

Finally, beginning this year and in alignment with European regulations, the China RoHS Regulation will restrict four phthalates—BBP, DBP, DEHP, and DIDP—to a maximum concentration of 0.1% by mass in electrical and electronic products [63] (Table 1).

**Table 1:** International regulatory restrictions on phthalate use.

Reference	Phthalate	Regulation	Country
[52]	BBP, DEHP, DINP, DIDP, DnOP	1999/815/EC: temporary restriction on children’s toys	European Union
[55]	BBzP, DEHP, DnBP	Directive 2005/84/EC: permanent restriction on children’s toys and articles	European Union
[56]	BBP, DBP, DEHP, DIBP	Commission Regulation (EU) 2018/2005: maximum concentration of 0.1% by weight in plasticized material in all plastic products	European Union
[57]	BBP, DBP, DEHP, DINP	Question numbers: EFSA-Q-2017-00588/-00589/-00590, EFSA-Q-2018-00800/-00801: TDI of 0.05 mg/kg body weight per day	European Union
[59]	DEHP	EU Regulation No. 2023/2482: restrictions to the use of DEHP in medical devices	European Union
[61]	DIDP	EPA-HQ-OPPT-2018-0435; EPA-HQ-OPPT-2024-0073: established risk for reproductive toxicity in female workers	United States
[62]	DINP	EPA-HQ-OPPT-2018-0436; EPA-HQ-OPPT-2024-0073: established risk for developmental toxicity, hepatotoxicity, and cancer at high exposure	United States
[63]	BBP, DBP, DEHP, DIDP	GB 26572-2025: maximum concentration of 0.1% by weight in electrical and electronic products	China

Abbreviations: BBP: butyl benzyl phthalate; BBzP: butylbenzyl phthalate DBP: di-n-butyl phthalate; DEHP: 2-ethylhexyl phthalate; DIBP: Diisobutyl phthalate; DIDP: di-iso-decyl phthalate; DINP: diisononyl phthalate; DnOP: di-n-butyl phthalate.

## 3. Phthalate Exposure and Ischemic Heart Disease: Epidemiological Evidence

Although not yet fully elucidated, emerging evidence suggests a potential risk of IHD associated with phthalate exposure (Table 2). As discussed in Section 1, IHD develops from atherosclerosis, a chronic inflammatory condition affecting large- and medium-sized arteries [64]. A Swedish cross-sectional study was the first to examine the association between ten circulating phthalate metabolites and four markers of atherosclerosis measured in both carotid arteries [65]. Among the 1016 participants aged 70 years, four of the ten metabolites—mono-isobutyl phthalate (MiBP, metabolite of DIBP), mono-methyl phthalate (MMP, metabolite of DMP), mono-ethyl phthalate (MEP, metabolite of DEP), and mono-(2-ethylhexyl) phthalate (MEHP, metabolite of DEHP)—were detectable in serum in all but 12 individuals [65]. Overall, 33.8% of participants exhibited unilateral carotid plaque, while 26.9% had bilateral plaque [65]. MMP levels were significantly associated with carotid plaques in an inverted U-shaped manner, even after adjustment for multiple risk factors. This finding suggests that DMP—primarily used in cosmetics and personal care products—may exert a direct atherosclerotic effect not mediated by traditional determinants of atherosclerosis (e.g., HDL- and LDL-cholesterol, serum triglycerides, smoking) [65,66]. Non-monotonic dose–response relationships, which may arise from opposing effects mediated by receptors with different affinities, receptor desensitization, or negative feedback at higher doses, have been reported for several chemicals, including phthalates, bisphenol A, polychlorinated biphenyls, pesticides, and dioxins, particularly in relation to their endocrine activity [67,68]. The observation of a similar pattern in atherosclerosis further supports the hypothesis that phthalates may exert a genuine effect on cardiovascular health [65]. MEP, MiBP, and MMP levels were also significantly and positively associated with the echogenicity (gray-scale media, GSM) of the intima–media complex (IM-GSM), and for MiBP and MMP also with plaque GSM, whereas MEHP showed an inverse association [65]. Notably, the GSM of the intima–media complex in the common carotid artery is closely related to the echogenicity of overt plaques [69]. Moreover, IM-GSM is associated with cardiovascular risk markers such as dyslipidemia, inflammation, and oxidative stress, which differ from traditional risk factors (BMI, blood pressure, smoking) that are linked to increased carotid intima–media thickness (IMT) [70]. IMT is a well-established and reliable predictor of carotid plaque prevalence; however, it has the limitation of including the media, a vessel layer that does not change in size during atherosclerosis progression [70,71]. Interestingly, none of the phthalate metabolites, except MMP, showed a significant inverse association with IMT, suggesting that phthalates may be more strongly involved in processes occurring within the intima alone rather than across both the intima and media layers [65].

A further investigation by Olsén and colleagues [71] examined the association between circulating levels of selected phthalate metabolites—MEP, MiBP, MEHP, and MMP—and coronary risk, as assessed by the Framingham Risk Score (FRS), in 1016 Swedish adults aged 70 years. The FRS incorporates six traditional risk factors, e.g., age, sex, HDL and total cholesterol, hypertension, and smoking, to estimate an individual’s 10-year risk of developing coronary heart disease (CHD) [72]. Serum levels of MEHP and MMP were significantly associated with LDL-cholesterol, MEP with diastolic blood pressure, MiBP with fasting glucose, and MMP with smoking [71]. However, despite these associations with individual CHD risk factors, only MMP showed a potential, though not statistically significant, association with the overall FRS [71].

A subsequent study [73] conducted in the same Swedish cohort investigated whether serum levels of monobenzyl phthalate (MBzP)—a metabolite of BBP, a phthalate widely used as a plasticizer in the polyvinyl chloride industry and also present in food conveyor belts, vinyl gloves, and adhesives [74]—were associated with markers of carotid atherosclerosis. MBzP showed a significant positive association with IMT, both as a continuous and categorical variable, even after adjustment for multiple cardiovascular risk factors [73]. It was also inversely and independently associated with both IM-GSM and plaque GSM, suggesting that higher MBzP levels may correspond to increased lipid infiltration within the vascular wall [73]. Similar to MMP, MBzP was associated with IMT but not with the number of overt atherosclerotic plaques, indicating a potential role in the early stages of atherosclerosis [65,73].

Overall, discrepancies within and between the two studies may reflect the weaker associations observed for GSM, since this parameter can only be assessed in individuals with plaques, whereas IM-GSM can be evaluated in nearly all participants [73]. They may also indicate that different phthalates exert distinct effects on the vascular wall [73].

Another cross-sectional study involving 793 Taiwanese adolescents and young adults aged 12–30 years—with and without childhood elevated blood pressure (EBP)—selected from an annual urine screening program examined the association between urinary DEHP metabolite levels and circulating endothelial microparticles (EMPs: CD62E and CD31^+^/CD42a^−^) and platelet microparticles (PMPs: CD62P and CD31^+^/CD42a^+^) [75]. These microparticles, vesicles measuring 0.1–1 µm in diameter, originate from cell apoptosis and inflammatory processes implicated in atherosclerosis development and serve as surrogate biomarkers of endothelial injury and vascular pathology [76,77]. The study also assessed CD14, a pattern-recognition receptor central to innate immunity and considered a potential risk factor for CHD [78]. Among the DEHP metabolites measured—MEHP, mono(ethyl-5-hydroxyhexyl) phthalate (MEHHP), and mono(2-ethyl-5-oxohexyl) phthalate (MEOHP)—MEHP was the only one to show a significant positive association with serum levels of CD31^+^/CD42a^−^, CD31^+^/CD42a^+^, and CD14, even after adjustment for age, sex, and cardiovascular risk factors including BMI, systolic blood pressure, LDL cholesterol, triglycerides, HOMA-IR, and smoking [75]. Specifically, interquartile increases in urinary MEHP were associated with 6.68% and 3.65% increases in EMPs and PMPs, respectively [75]. Importantly, urinary MEHP alone was significantly associated with higher BMI and HOMA-IR, with younger and female participants exhibiting higher concentrations, likely reflecting greater use of cosmetics and other DEHP-containing consumer products [75]. CD31^+^/CD42a^+^ levels increased significantly with HOMA-IR, whereas CD31^+^/CD42a^−^ was positively associated with BMI, LDL cholesterol, triglycerides, HOMA-IR, and systolic blood pressure, and inversely associated with HDL cholesterol [75]. Collectively, these findings suggest that MEHP may contribute to atherosclerosis by preferentially promoting endothelial and platelet apoptosis, potentially through a reactive oxygen species (ROS)-mediated mitochondria-dependent pathway, followed by inflammatory activation (see Section 4) [75,79]. This cascade may increase endothelial monolayer permeability, facilitating smooth muscle cell (SMC) proliferation and migration, as well as the infiltration of lipids and monocytes into the intima, ultimately contributing to plaque development and the onset of atherosclerosis [75,80].

From the same nationwide urine screening program for renal health among Taiwanese schoolchildren aged 6–18 years, Su et al. recruited 789 participants—38.4% with EBP—to assess the association between seven urinary phthalate metabolites (MMP, MEP, MBzP, MEHP, MEHHP, MEHOP, and mono-n-butyl phthalate [MnBP]) and IMT [81]. Consistent with previous findings [73], urinary MEHP levels were positively associated with major cardiovascular risk factors, including BMI, waist circumference, triglycerides, diastolic blood pressure, and the prevalence of diabetes and hypertension [81]. Multiple linear regression analyses revealed a dose–response relationship between mean and maximal carotid IMT across different carotid segments and log-transformed urinary levels of MEHP, MnBP, and the sum of DEHP metabolites (∑DEHP: MEHP, MEHHP, MEHOP), after adjustment for age, sex, BMI, C-reactive protein (CRP), biochemical indicators (fasting glucose, LDL-cholesterol, triglycerides), childhood EBP, physical activity, smoking and alcohol habits, and socioeconomic status. These findings provide evidence of a dose–response relationship between phthalate exposure and subclinical atherosclerosis in young populations [81]. Additionally, multivariate regression analysis showed that individuals in the highest quartile of urinary MEHP had a 7.39-fold increased risk of elevated carotid IMT (95% CI: 4.16–13.12) compared with those in the lowest quartile [81]. ∑DEHP and MnBP exhibited similar, though less pronounced, associations, with increased risks of 2.80 (95% CI: 1.65–4.75) and 2.46 (95% CI: 1.46–4.14), respectively [81].

A subsequent cross-sectional study involving 783 Taiwanese participants aged 12–30 years examined the relationship between urinary concentrations of DEHP metabolites, carotid IMT, and global DNA methylation levels—quantified as the ratio of 5-methyl-2-deoxycytidine (5mdC) to 2-deoxyguanine (dG)—given the potential involvement of genomic DNA methylation in the early stages of CVD pathogenesis [82,83,84]. Supporting this hypothesis, a large longitudinal, multi-cohort epigenome-wide investigation including 11,461 participants identified methylation at 52 cytosine-phosphate-guanine (CpG) sites as being associated with increased risk of CHD and AMI events [85]. In the study by Lin et al. [82], MEHP was positively associated with HOMA-IR, consistent with previous findings [75,81]. Global methylation levels, assessed in leukocytes from 71.8% of participants, were significantly and positively correlated with both HOMA-IR and carotid IMT, but not with other CVD risk factors [82]. Multiple linear regression analyses showed that urinary MEHP levels were significantly and positively associated with both 5mdC/dG and carotid IMT after adjustment for age, sex, BMI, smoking status, systolic blood pressure, HOMA-IR, triglycerides, and LDL cholesterol, whereas MEHHP and MEOHP showed no significant associations [82]. The association between MEHP and carotid IMT was particularly strong when carotid thickness exceeded the 75th percentile (IR = 1.40, *p* < 0.001) [82]. Similarly, the association between MEHP and 5mdC/dG was stronger above the median (OR = 1.44, *p* < 0.001) than below it (OR = 1.13, *p* = 0.155) [81]. Structural equation modelling further indicated that MEHP was positively associated with both carotid IMT and 5mdC/dG (*p* < 0.001), and that 5mdC/dG was itself positively associated with IMT [82]. These findings suggest that MEHP exerts both direct and indirect effects on carotid IMT, with global DNA methylation acting as a mediator in DEHP-induced atherosclerosis, although the lack of locus-specific methylation data limits mechanistic interpretation (see Section 4) [82].

Using data from the 2003–2004 National Health and Nutrition Examination Survey (NHANES), a population-based survey assessing health and nutritional status across the United States, Zhang et al. [32] evaluated the association between phthalate exposure—measured through urinary levels of 11 metabolites—and high-sensitivity cardiac troponin I (hs-cTnI) in 2241 subjects. This assay detects minimal increases in circulating cardiac troponin, a highly specific biomarker of myocardial injury, in more than 50% of asymptomatic individuals, with a coefficient of variation below 10% at the 99th percentile in healthy subjects, making it a robust tool for cardiovascular risk stratification [86]. Notably, non-ischemic myocardial injury is the most common cause of elevated hs-cTnI and is associated with poor prognosis in nearly 75% of affected individuals [87]. Zhang and co-workers reported significant increases in hs-cTnI levels (3.4–4.0%) associated with a one-standard-deviation increase in urinary concentrations of ∑DEHP (MEHP, MECPP, MEHHP, MEOHP), ∑LMWP (MMP, MEP, MBP, MiBP), ∑HMWP (∑DEHP, MCPP, MBzP), as well as MECPP and MEP, after adjusting for BMI, glycated hemoglobin, HDL and total cholesterol, systolic blood pressure, and estimated glomerular filtration rate (eGFR) [32]. Marked heterogeneity was observed across sex and age groups: ∑LMWP and MEP were strongly associated with hs-cTnI among female children and adolescents (6–19 years), whereas ∑HMWP, ∑DEHP, and individual DEHP metabolites were significantly associated with hs-cTnI in male adults aged ≥20 years, suggesting age- and sex-specific susceptibility to the endocrine and metabolic effects of phthalates [32]. Importantly, co-exposure to phthalate mixtures was also significantly and positively associated with hs-cTnI (5.6% increase per one-unit rise in the weighted quantile sum), with MECPP contributing most strongly, followed by MMP and MEP [32]. Given the significant associations of ∑HMWP, ∑DEHP, and their metabolites, except MEP, with BMI, HOMA-IR, and eGFR, these findings suggest that obesity, insulin resistance, and hyperglycemia may mediate the potentially cardiotoxic effects of phthalates on the myocardium [32].

Su et al. were the first to investigate the relationship between urinary concentrations of phthalate metabolites and confirmed CHD in a case-control study including 180 subjects randomly selected from 336 CHD patients under 60 years of age, along with 360 age- and sex-matched non-CHD controls [88]. Stratification of CHD patients by discharge date after diagnosis revealed that those who were hospitalized and immediately discharged exhibited the highest geometric mean urinary levels of MEHP, MEHHP, MEHOP, and ∑DEHP, followed by patients discharged within fewer than 3 days, and finally those discharged 3 days or more after diagnosis [88]. This pattern suggests elevated DEHP exposure among hospitalized patients, likely due to its extensive use in medical devices, as discussed in Section 2 [88]. In contrast, MEP levels were significantly higher in subjects discharged on or after the third day compared with those discharged earlier [88]. Notably, LMW phthalates such as DEP and DBP are used as coatings for certain oral medications (e.g., stool softeners and laxatives), which can result in urinary phthalate metabolite concentrations up to ten times higher among users than non-users [89]. When restricting the analysis to CHD patients discharged on or after the third day, creatinine-adjusted levels of MEHP, MnBP, and MiBP were significantly higher in patients than in controls [88]. After adjustment for age, sex, BMI, hypercholesterolemia, hypertension, T2D, statin use, smoking, and alcohol consumption, individuals in the highest tertiles of MEHP (Odds Ratio—OR = 2.77, 95% CI: 1.22–6.28, *p* = 0.021) and MiBP (OR = 3.19, 95% CI: 1.41–7.21, *p* = 0.010) had a significantly higher risk of CHD compared with those in the lowest tertiles [88]. Furthermore, levels of hs-CRP, fibrinogen, and D-dimer increased significantly across quartiles of urinary concentrations of individual DEHP metabolites and ∑DEHP, with the exception of fibrinogen, which showed no association with MEHP [88]. These biomarkers are key contributors to atherosclerosis. Hs-CRP, a marker of chronic inflammation, plays a central role across all stages of atherosclerosis and correlates strongly with disease burden [90]. Fibrinogen participates in inflammation, intercellular interactions, and cell migration; elevated levels promote macrophage, leukocyte, and platelet recruitment, contributing to plaque formation, SMC proliferation, and angiogenesis, making it a critical ASCVD risk factor [91]. Similarly, D-dimer, a degradation product of cross-linked fibrin reflecting activated coagulation and fibrinolysis, has been associated with ischemic cardiovascular events, including AMI and the severity of coronary artery disease [92]. In this context, the significant associations between urinary DEHP metabolites, CHD, and elevated inflammatory and thrombosis markers further support a potential role for phthalates in the etiopathogenesis of atherosclerosis [88]. However, the authors acknowledged that increases in inflammatory markers may partly reflect acute or subacute events directly related to CHD itself [88].

In summary, phthalates—particularly DEHP, and to a lesser extent DMP and DiBP—may exert detrimental effects on vascular health either by directly altering carotid IMT or through interconnected mechanisms involving hyperglycemia, dyslipidemia, pre-diabetic metabolic disturbances, endothelial and platelet apoptosis, and changes in global DNA methylation. Phthalate exposure also appears to be associated with the development and severity of IHD by promoting inflammation, atherosclerotic plaque formation, and thrombosis. Of note, differences between studies in the associations with IHD may depend on the specific phthalate evaluated, as LMW and HMW phthalates differ substantially in their rates of metabolism and excretion (Table 1).

Despite these insights, the cross-sectional design of available studies limits causal inference. In addition, published investigations relied on a single measurement of phthalate levels, which, although widely accepted for exposure assessment, presents important limitations. Because phthalates are rapidly metabolized and excreted (typically within 1–2 days) a single serum or urine sample reflects only recent exposure to the parent compound or its metabolites, with the specific time window varying by phthalate. Consequently, this approach may not adequately capture long-term exposure patterns, particularly in the general population, and is also subject to variability depending on the time of day at which the sample is collected. While the sensitivity and specificity of urinary biomarkers allow for a reasonable estimation of exposure across short time windows—extending in some cases to several months—repeated measurements over time would substantially improve the reliability of exposure assessment when investigating chronic outcomes such as IHD. This underscores the urgent need for prospective, large-scale investigations to validate these preliminary observations and clarify the long-term cardiovascular consequences of phthalate exposure.

**Table 2:** Key findings and research gaps on phthalate exposure and ischemic heart disease.

Reference	Pitfalls	Reference	Clues
[65]	No significant association between circulating MEP levels and plaque GSM	[65]	Serum MMP levels significantly related to the number of plaques in an inverted U-shaped manner
[65]	No significant association of MEHP, MEP, and MiBP in serum with IMT	[65,73]	Serum MMP and MBzP levels significantly and inversely associated with IMT
[71]	No significant association between serum levels of MEP, MEHP, MiBP, and MMP and FRS	[65,73]	MBzP, MiBP, and MMP in serum significantly and positively associated with IM-GSM and plaque GSM
[73]	Serum MBzP levels not significantly related to plaque prevalence	[65]	MEP in serum significantly and positively associated with IM-GSM
[32,65,71,73,75,81,82,88]	Cross-sectional design	[65]	Serum MEHP concentration inversely related to IM-GSM and plaque GSM
[32,75,81,82,88]	Single-spot urine measurement	[71]	Circulating levels of MEHP and MMP associated with LDL cholesterol, MEP with diastolic blood pressure, and MiBP with fasting glucose
[65,71,73]	Study performed exclusively on Caucasian individuals aged 70	[75]	Urinary MEHP concentration significantly and positively associated with serum levels of CD31^+^/CD42a^−^, CD31^+^/CD42a^+^, and CD14
[75]	No significant association of urinary MEHHP and MEHOP levels with EMPs and PMPs	[81]	Urinary MEHP, MnBP, and ∑DEHP levels significantly and positively associated with both maximal and mean values of carotid IMT as both continuous and categorical variables
[75]	Study conducted on adolescents and young adults with abnormal urinalysis	[82]	Urinary MEHP levels significantly and positively correlated with 5mdC/dG and carotid IMT
[75,81,82,88]	No adjustment of associations for medications, genetics and other occupational environmental factors (e.g., bisphenol A, perfluorooctane sulfate, air pollutants)	[32]	Significantly positive association of urinary levels of ∑DEHP, ∑LMWP, ∑HMWP, MEP, and MECPP significantly with hs-cTnI
[81]	Urinary concentration of MEHHP and MEHOP not significantly associated with carotid IMT	[32]	Phthalate co-exposure significantly associated with an increase in hs-cTnI percentage
[82]	MEHHP and MEHOP not significantly associated either with carotid IMT or 5mdC/dG	[88]	Highest tertiles of urinary MEHP and MiBP significantly associated with increased risk of CHD
[82]	Study performed exclusively on young adults	[88]	Increased serum concentration of hsCRP and D-dimer significantly and positively associated across tertiles of urinary ∑DEHP, MEHP, MEHHP, and MEHP
[82]	No measurement of methylation at specific gene loci	[88]	Increased serum concentration of fibrinogen significantly and positively associated across tertiles of urinary ∑DEHP, MEHHP, and MEHP
[32]	No significant association of urinary concentration of MEHP, MEHHP, MEHOP, MCPP, MBzP, MMP, MBP, and MiBP, with hs-cTnI		
[32]	Underestimation of hs-cTnI due to its degradation if long stored under −80 °C		
[88]	No significant association of urinary MEHHP, MEHOP, ΣDEHP, MMP, MnBP, MBzP, MiBP with increased risk of CHD		

Abbreviations: 5mdC/dG: ratio of 5-methyl-2′-deoxycytidine (5mdC) to deoxyguanosine (dG); ∑DEHP: sum of MEHP, MEHHP, and MEHOP; ∑HMWP: sum of ∑DEHP, MCPP, and MBzP; ∑LMWP: sum of MMP, MEP, MBP, and MiBP; CHD: coronary heart disease; EMP: endothelial microparticle; FRS: Framingham Risk Score; GSM: gray scale media; hs-cTnI: high-sensitivity cardiac troponin I; IMT: intima–media thickness; LDL: low-density lipoprotein; MBzP: monobenzyl phthalate; MEHHP: mono(ethyl-5-hydroxyhexyl) phthalate; MEHOP: mono(2-ethly-5-oxoheyl) phthalate; MEHP: mono (2 ethylhexyl) phthalate; MEP: mono-ethyl phthalate; MiBP: mono-isobutyl phthalate; MMP: mono-methyl phthalate; MnBP: mono-n-butyl phthalate; PMP: platelet microparticle.

## 4. Cellular and Molecular Mechanisms Underlying Phthalate-Induced Ischemic Heart Disease

Exposure to phthalates induces remarkable cellular and molecular alterations that involve pathways, organelles and macromolecules crucial for cellular physiology and viability. These imbalances and modifications significantly contribute to the onset of a plethora of diseases, including IHD. The main alterations are described in the following paragraphs, and the mechanistic studies are summarized in Table 3.

### 4.1. Oxidative Stress, Inflammation, and Apoptosis

The interplay between oxidative stress, inflammation, and apoptosis represents a hierarchical and self-amplifying pathophysiological cascade central to CVD onset and progression [93]. Increasing in vitro studies demonstrate that phthalates and their metabolites could trigger one or more steps of this cascade. Interestingly, Ban et al. demonstrated that MEHP exposure leads to a dose-dependent increase in intracellular ROS, depletion of the antioxidant glutathione (GSH), and elevation of malondialdehyde (MDA), a biomarker of lipid peroxidation, and ROS-induced membrane injury, indicating a substantial oxidative stress triggered by MEHP. This phenomenon results in loss of mitochondrial membrane potential, promoting cytochrome c release, and activating the intrinsic apoptotic pathway in human umbilical vein endothelial cells [79]. The intrinsic apoptotic cascade coming from the collapse of mitochondrial membrane potential can be promoted by cathepsin B release from lysosome, after the stimulation of autophagy [94], as result of the oxidative stress due to MEHP exposure in another model of endothelial cells [95]. The authors demonstrated that MEHP induces autophagy in EA.hy926 cells in a dose-dependent manner through the Akt1 pathway, leading to increased autophagosome formation and autophagic cell death, and in this process, ROS played an important role.

Increases in ROS and impairment of antioxidant systems were also observed in cardiomyocytes exposed to DEHP. This phthalate triggers inflammatory responses through upregulation of prostaglandin-endoperoxide synthase 2/cyclooxygenase 2, which promotes pro-inflammatory signaling, and simultaneously activates apoptotic pathways, resulting in increased cell death [96]. In addition, molecular docking and molecular dynamics simulation studies demonstrated that DEP and its metabolite MEP bind the antioxidant enzyme superoxide dismutase (SOD), inducing structural changes that destabilize the protein and inhibit its catalytic activity, thereby weakening cellular antioxidant defenses and increasing susceptibility to oxidative stress [97]. These findings offer valuable insights into the molecular binding mechanisms and structural implications for SOD, establishing a foundation for understanding the potential health risks related to oxidative stress and the toxicological regulation of PAEs and their metabolites.

**Table 3:** In vitro animal and human studies supporting the correlation between phthalate exposure and IHD-promoting conditions.

Oxidative Stress, Inflammation, and Apoptosis				
References	Methods	Models and Treatments	Biological Meaning	Identified/Described Mechanisms
[79]	Intracellular ROS measurement (DCFH-DA fluorescence assay) GSH content determination (colorimetric assay) Lipid peroxidation assessment via MDA levels (TBARS assay) Mitochondrial membrane potential (ΔΨm) analysis (JC-1 staining) Cytochrome c release (Western blot) Apoptosis assessment (Annexin V/PI flow cytometry) Caspase-9 and caspase-3 activation (Western blot/activity assays)	In vitro model: Human umbilical vein endothelial cells (HUVECs) Treatment: MEHP exposure at 0, 6.25, 12.5, 25, 50, and 100 μM; 24 h	Oxidative stress-mediated lipid peroxidation and mitochondrial damage leading to activation of the intrinsic apoptotic pathway in endothelial cells	MEHP → ↑ intracellular ROS, ↓ GSH, ↑ MDA → mitochondrial dysfunction → apoptosis
[94]	Autophagy assessment (LC3-I/LC3-II conversion, Beclin-1 expression; Western blot) Autophagosome formation (GFP-LC3 fluorescence microscopy) Lysosomal membrane permeabilization (LysoTracker staining) Cathepsin B release and activity (Western blot and enzymatic assay) Mitochondrial membrane potential (ΔΨm) analysis (JC-1 staining) Cytochrome c release (Western blot) Apoptosis quantification (Annexin V/PI flow cytometry) Caspase-9 and caspase-3 activation (Western blot) Pharmacological inhibition of autophagy and cathepsin B to confirm pathway involvement	In vitro model: Human endothelial cells (HUVECs) Treatment: MEHP exposure at 0, 25, 50, 100, and 200 μM; 6, 12, and 24 h	Autophagy-dependent lysosomal–mitochondrial crosstalk promotes endothelial apoptosis via activation of the intrinsic apoptotic pathway	MEHP → autophagy activation → lysosomal destabilization → cathepsin B release → mitochondrial dysfunction → intrinsic apoptosis
[95]	Intracellular ROS measurement (DCFH-DA fluorescence assay) Autophagy markers analysis (LC3-I/LC3-II conversion, Beclin-1 expression; Western blot) Autophagosome formation (transmission electron microscopy; fluorescence microscopy) Akt1 pathway analysis (Akt1 phosphorylation status; Western blot) Pharmacological modulation of ROS (antioxidant pretreatment) Cell viability and autophagic cell death assays (MTT assay, LDH release)	In vitro model: Human vascular endothelial cells (EA.hy926) Treatment: MEHP exposure at 0, 25, 50, 100, and 200 μM; 24 h	ROS-mediated Akt1 signaling disruption promotes excessive autophagy and autophagic cell death in endothelial cells	MEHP → ↑ ROS → Akt1 pathway inhibition → autophagy activation → autophagic cell death
[96]	Intracellular ROS measurement (fluorescent ROS probes) Antioxidant system evaluation (GSH content, antioxidant enzyme activity assays) Lipid peroxidation assessment (MDA levels) Inflammatory pathway analysis (PTGS2/COX-2 expression; Western blot and qPCR) Pro-inflammatory cytokine measurement (ELISA) Apoptosis assessment (Annexin V/PI staining; caspase-3 activation) Cell viability assays (CCK-8/MTT)	In vitro model: Primary rat cardiomyocytes Treatment: DEHP exposure at 0, 25, 50, 100, and 150 μM; 24, 48 h	Oxidative stress-driven inflammatory signaling promotes cardiomyocyte injury and apoptotic cell death	DEHP → ↑ ROS, ↓ antioxidant defenses → PTGS2 (COX-2) upregulation → inflammation → apoptosis
[97]	Molecular docking analysis (DEP–SOD and MEP–SOD binding affinity and interaction sites) Molecular dynamics simulations to assess protein structural stability Analysis of conformational changes (RMSD, RMSF, radius of gyration) Evaluation of catalytic site perturbation and enzyme flexibility	In silico model: Human superoxide dismutase (SOD) structure Treatment: Molecular interaction with DEP and its metabolite MEP	Direct inhibition of antioxidant enzyme activity compromises cellular redox homeostasis, favoring oxidative stress	DEP/MEP → direct binding to SOD → structural destabilization → ↓ SOD catalytic activity → ↑ oxidative stress susceptibility
[98]	Cardiac oxidative stress evaluation (MDA levels; protein carbonyl content assays) Lipid peroxidation assessment (TBARS assay) Serum lipid profile analysis (total cholesterol, triglycerides, LDL-C, HDL-C) Atherogenic index calculation Histopathological analysis of cardiac tissue	Animal model: BALB/c mice (male) Treatment: Intraperitoneal administration of DEHP at 5, 50, and 200 mg/kg body weight daily for 30 consecutive days	Oxidative stress-induced cardiac damage and lipid metabolism alteration increase atherosclerosis and cardiovascular risk	DEHP → ↑ cardiac ROS, ↑ lipid peroxidation → dyslipidemia → ↑ atherogenic index
[99]	Serum lipid profile (total cholesterol, LDL-C, HDL-C) Atherosclerotic lesion analysis (Oil Red O staining of aortic root and en face aorta) Endothelial inflammation markers (VCAM-1, ICAM-1; Western blot, immunohistochemistry) Macrophage infiltration in plaques Oxidative stress and inflammatory signaling evaluation	Animal model: Apolipoprotein E-deficient mice (ApoE^−^/^−^, male) Treatment: Oral administration of DEHP at 1500 mg/kg/day via drinking water for 4 weeks	Chronic DEHP exposure promotes atherogenesis via lipid dysregulation and vascular inflammation	DEHP → cholesterol homeostasis disruption + endothelial inflammation → accelerated atherosclerosis
[100]	Histopathology (H&E staining; assessment of disorganized myocardial fibers, collagen deposition) Serum cardiac injury markers (CK-MB, CRP; ELISA) Pyroptosis assessment (caspase-1 activation; GSDMD cleavage; Western blot) Sphingolipid pathway analysis (SPHK1, S1PR2 expression; qPCR and Western blot) Pharmacological inhibition of SPHK1/S1PR2 to confirm pathway involvement	Animal model: Male C57BL/6J mice Treatment: Oral administration of DEHP at 500 mg/kg/day via gavage for 28 consecutive days	Disruption of sphingolipid metabolism triggers inflammatory programmed cell death, leading to myocardial injury	DEHP/MEHP → SPHK1/S1PR2 sphingolipid pathway activation → cardiomyocyte pyroptosis → cell death
[101]	Transcriptomic analysis for apoptotic gene expression (Bcl-2 family, Fas/FasL) Flow cytometry for apoptosis quantification (Annexin V/PI) Caspase activity assays (caspase-3, caspase-9) ROS measurement (DCFH-DA fluorescence assay) Evaluation of antioxidant status (GSH, SOD activity)	Animal model: Sprague-Dawley rats (male) Treatment: Intragastric (oral gavage) administration of DMP at 0, 50, 100, and 200 mg/kg/day	Oxidative stress-mediated activation of multiple apoptotic cascades promotes cardiomyocyte death	DMP → ↑ ROS → intrinsic (Bax/Bcl-2) & extrinsic (Fas/FasL) apoptotic pathways → Caspase-dependent cardiomyocyte apoptosis
[102]	NLRP3 inflammasome assessment (NLRP3, ASC, caspase-1 expression; Western blot) IL-1β and IL-18 quantification (ELISA) Histology & immunohistochemistry for neutrophil and macrophage infiltration Cardiac injury evaluation (histopathology, infarct size measurement)	Animal model: Male C57BL/6N mice Treatment: Acute exposure to DEHP via intraperitoneal injection at 30 mg/kg/day for 7 days during the recovery period following surgically-induced myocardial infarction	Pro-inflammatory signaling and innate immune activation exacerbate myocardial injury	Phthalates → NLRP3 inflammasome activation → ↑ IL-1β, ↑ IL-18 → neutrophil & macrophage infiltration → cardiac inflammation
[103]	Lipid peroxidation assessment (MDA assay; TBARS) Mitochondrial damage evaluation (TEM; mitochondrial membrane potential assay) Ferroptosis markers (GPX4 expression, ACSL4, iron accumulation; Western blot, biochemical assays) Nrf2/HO-1 pathway analysis (Western blot, qPCR, immunohistochemistry) ROS measurement (DCFH-DA fluorescence)	Animal model: Male C57BL/6J mice Treatment: Oral gavage administration of DEHP at 50, 200, and 500 mg/kg body weight per day for 28 consecutive days.	Oxidative stress-induced iron-dependent cell death contributes to cardiomyocyte injury	DEHP → ↑ lipid peroxidation + mitochondrial damage → Nrf2/HO-1 pathway activation → ferroptosis in cardiomyocytes
[104]	Oxidative stress measurement (ROS assay, MDA/TBARS, GSH levels) Mitochondrial function assays (mitochondrial membrane potential, morphology via TEM) Inflammatory & pyroptosis markers (NLRP3, caspase-1, GSDMD; Western blot, immunohistochemistry) Lipid profile & endocrine parameter evaluation (serum cholesterol, triglycerides, hormones) Cardiac histopathology (fibrosis assessment; Masson’s trichrome staining) Functional assessment (echocardiography, if included)	Animal model: Sprague-Dawley rats (male) Treatment: Oral gavage administration of DBP at 0.01, 1, and 50 mg/kg/day for 12 weeks combined with high-fat diet for sub-chronic period	Oxidative stress, inflammation, pyroptosis, and metabolic/endocrine disruption collectively promote structural myocardial damage and cardiotoxicity	DBP + high-fat diet → ↑ ROS, ↑ MDA, ↓ GSH → membrane & mitochondrial damage → activation of NLRP3 inflammasome, caspase-1, GSDMD → inflammatory + pyroptotic pathways → disrupted lipid metabolism & endocrine homeostasis → cardiac fibrosis & dysfunction
[105]	Microparticle isolation and quantification (flow cytometry, annexin V labeling) Tissue factor expression analysis (Western blot, qPCR) Signaling pathway assessment (TGF-β1/Smad/PAI-1; Western blot, inhibitor studies) Functional coagulation assays (procoagulant activity of microparticles)	In vitro model: Human M1 macrophages Treatment: DEHP at 0, 10, 50, and 100 μM; 24 h	Vascular inflammation and enhanced thrombogenic potential contribute to CVD progression	DEHP → TGF-β1/Smad/PAI-1 pathway activation in M1 macrophages → ↑ tissue factor–bearing microparticle release → procoagulant activity
[106]	Urinary phthalate metabolite quantification (LC-MS/MS) Oxidative stress biomarkers in urine/plasma (8-OHdG, MDA; ELISA/HPLC) Blood pressure measurement (systolic/diastolic) Statistical correlation analysis (regression models, adjustment for confounders)	Human cohort: >1000 individuals Treatment: No experimental treatment; observational study	Phthalate-induced oxidative stress contributes to cardiovascular risk and elevated blood pressure in humans	Urinary phthalate metabolites (MEP, MBP, MiBP, MBzP, sum of six) → ↑ oxidative stress biomarkers (8-OHdG, MDA) → ↑ blood pressure & hypertension prevalence
[35]	Urinary MEHP quantification (LC-MS/MS) Oxidative stress markers (MDA, SOD activity; ELISA/biochemical assays) Correlation/statistical analysis with CHD status	Human case-control study: CHD patients and matched controls Treatment: No experimental treatment; observational study	Oxidative stress contributes to CHD pathology	Urinary MEHP → ↑ MDA, ↓ SOD → oxidative stress associated with CHD
[75]	Urinary phthalate metabolite measurement (LC-MS/MS) Microparticle identification & quantification (flow cytometry: CD31, CD42a, CD14 markers) Correlation/statistical analysis between urinary metabolites and microparticle levels	Human cohort: Adolescents and young adults Treatment: No experimental treatment; observational study	Microparticle release reflects apoptosis and inflammation contributing to atherosclerosis	Urinary MEHP/DEHP metabolites → ↑ endothelial (CD31+/CD42a−) & platelet (CD31+/CD42a+) microparticles → activation of monocytes, macrophages, neutrophils (CD14)
[88]	Urinary DEHP metabolite measurement (LC-MS/MS) Serum inflammatory and thrombotic marker quantification (hs-CRP, fibrinogen, D-dimer; ELISA/clinical assays) Statistical correlation analysis	Human case-control study: CHD patients Treatment: No experimental treatment; observational study	Phthalate-induced oxidative/inflammatory stress promotes pro-atherothrombotic state in CHD patients	Urinary DEHP metabolites → ↑ hs-CRP, fibrinogen, D-dimer → vascular inflammation & thrombosis
[33]	Pathway enrichment analysis (PI3K-Akt, JAK-STAT, BCL2, PIK3CA) Protein–protein interaction network construction Molecular docking simulations (binding affinity between phthalates and target proteins) Integration with NHANES biomarker/exposure data	In silico + human data: NHANES 2005–2018 dataset; computational network toxicology analysis Treatment: No experimental treatment; observational/computational study	Oxidative stress, inflammation, and apoptosis drive cardiovascular toxicity in cardiac and vascular cells	Phthalate metabolites → binding to PI3K-Akt, JAK-STAT, BCL2, PIK3CA → oxidative stress, inflammatory signaling, apoptosis
**Mitochondrial DNA Alterations and Dysfunction**				
**References**	**Methods**	**Models and Treatments**	**Biological Meaning**	**Identified/Described Mechanisms**
[34]	ROS measurement (MitoSOX, DCFDA) Antioxidant enzyme activity (SOD, GSH) Mitochondrial membrane integrity (JC-1, TMRE) mtDNA copy number quantification (qPCR) Mitochondrial dynamics & biogenesis markers (Western blot, immunofluorescence) Oxidative phosphorylation assessment (Seahorse XF, ATP assays)	In vitro models: Human endothelial cells and cardiomyocytes Treatment: DEHP from 10 to 100 μM; 24, 48 h Animal model: C57BL/6 mice Treatment: DEHP by oral gavage at 50, 200, and 500 mg/kg/day for 28 days	Mitochondrial redox imbalance and structural/functional impairment contribute to cardiomyocyte dysfunction and CVD risk	DEHP → ↑ mitochondrial ROS → oxidative damage to membranes, proteins, mtDNA; disrupted mitochondrial dynamics & biogenesis → defective oxidative phosphorylation, ↓ mtDNA copy number
[30]		In vitro model: Primary human vascular endothelial cells Treatment: DEHP at 10, 50, and 100 μM; 24 h Animal model: Sprague-Dawley rats Treatment: DEHP at 100 and 400 mg/kg/day via oral gavage for 12 weeks		
[107]	Mitochondrial morphology (fluorescence microscopy, Mitotracker staining) Mitochondrial membrane potential (ΔΨm; JC-1/TMRE) ATP content measurement (bioluminescence assay)	In vitro model: Zebrafish (Danio rerio) ZF4 cells Treatment: DBP exposure at 0, 10, 50, and 100 μM; 24 h	Collapse of mitochondrial bioenergetic capacity, contributing to cardiomyocyte/vascular cell dysfunction	DBP → mitochondrial fragmentation, ↓ ΔΨm, ↓ ATP synthesis
[108]	mtDNA damage assessment (long-amplicon qPCR, lesion quantification) mtDNA copy number quantification (qPCR) Cytosolic mtDNA measurement (PCR, imaging) cGAS-STING pathway activation (Western blot, immunofluorescence for cGAS, STING, downstream cytokines)	Animal model: Zebrafish (Danio rerio) embryos and larvae Treatment: DBP at 0.1, 1, and 10 μM in the aquatic medium; up to 7 days post-fertilization	mtDNA damage links mitochondrial dysfunction to cellular inflammation and contributes to cardiovascular risk	DBP → ↑ mtDNA lesions, ↓ mtDNA copy number → mtDNA release → cGAS-STING pathway activation
[109]	mtDNA copy number quantification (qPCR) Statistical association with cardiovascular outcomes	Human cohort: Leukocytes from >1000 individuals Treatment: Observational study, no experimental treatment	Reduced mtDNA integrity is linked to higher susceptibility to CHD and CVD	↓ mtDNA copy number → ↑ risk of atherosclerosis and major cardiovascular events
[110]	Meta-analysis of observational studies Risk ratio (RR) calculation for CVD, CHD, HF, stroke, all-cause mortality	Human observational studies: Multiple cohorts included in meta-analysis Treatment: Observational, no experimental intervention	mtDNA copy number is a robust biomarker of mitochondrial dysfunction and adverse cardiovascular outcomes	↓ mtDNA copy number → ↑ risk of CVD, CHD, HF, stroke, all-cause mortality
**DNA Methylation and Non-Coding RNAs**				
**References**	**Methods**	**Models and Treatments**	**Biological Meaning**	**Identified/Described Mechanisms**
[111]	miRNA expression (qPCR) MYOCD protein quantification (Western blot) VSMC phenotypic markers (immunofluorescence, α-SMA, SM22α for contractile; OPN, vimentin for synthetic) Functional assays for cell proliferation/migration (scratch assay, transwell)	In vitro model: A7r5 rat vascular smooth muscle cells (VSMCs) Treatment: DBP at 10 μM; 24 h	Promotion of atherosclerotic plaque formation and instability	DBP → ↑ miR-139-5p → ↓ myocardin (MYOCD) → VSMC phenotypic switch (contractile → synthetic)
[112]	miRNA expression (qPCR) SP1 and MCP-1 protein levels (Western blot, ELISA) Monocyte adhesion assay (fluorescent-labeled monocytes)	In vitro model: EA.hy926 human vascular endothelial cells Treatment: DBP at 10 μM; 24 h	Enhanced monocyte recruitment to endothelium, promoting early atherogenesis	DBP → ↓ miR-137-3p → ↑ SP1 → ↑ MCP-1
[113]	lncRNA and miRNA expression (qPCR) VSMC proliferation assay (BrdU, Ki-67 staining) Apoptosis assay (Annexin V/PI staining, caspase activity) Protein markers of VSMC phenotype (Western blot, α-SMA, SM22α)	In vitro model: RAW264.7 murine macrophages and rat vascular smooth muscle cells (VSMCs) Treatment: DEHP at 100 μM; 24 h	Promotion of vascular damage, plaque instability, and risk of coronary artery disease (CAD)	DEHP → ↑ GAS5 (lncRNA) → sequestration of miR-145-5p → ↑ VSMC proliferation + ↑ apoptosis
[114]	Genome-wide DNA methylation analysis (RRBS) Transcriptomic profiling (RNA-seq) Cell-type proportion inference Longitudinal epigenetic and gene expression analysis	Animal model: C57BL/6J mice (both sexes) Treatment: DEHP at 25 mg/kg chow or control chow, starting 2 weeks prior to mating and continuing through pregnancy and lactation until weaning at postnatal day 21	Early-life epigenetic reprogramming of the heart that persistently modifies gene expression patterns and cardiac cellular architecture, predisposing to increased cardiovascular risk later in life	Perinatal DEHP exposure → ↑ Differentially methylated regions (DMRs) and ↑ differentially expressed genes (DEGs) → persistent alteration of cardiac cell-type proportion and composition
[115]	RRBS (DNA methylation profiling) RNA-seq (gene expression) DMR and DEG analysis (sex-stratified)	Animal model: C57BL/6J mice (both sexes) Treatment: Oral, DEHP at 25 mg/kg chow beginning 2 weeks prior to mating, continuing through pregnancy and lactation until weaning at postnatal day 21	Developmental DEHP exposure programs long-lasting, sex-dependent alterations in cardiac gene regulation, potentially increasing later-life CVD risk	DEHP (developmental exposure) → ↑ DMRs → persistent cardiac epigenetic reprogramming (sex-specific)
[116]	Urinary phthalate metabolites: LC–MS/MS Blood pressure measurement in children: standardized sphygmomanometry DNA methylation analysis: Bisulfite conversion + targeted methylation assays (hypertension-related genes, e.g., ECE1, SCNN1G) Mediation analysis: Statistical modeling	Human study: Pregnant women (n = 198) during 3rd trimester and their preschool-age children Treatment: No experimental treatment; observational exposure to phthalates (MMP, MEP, MEcPP)	Prenatal phthalate exposure epigenetically programs hypertension risk, linking early-life exposure to later cardiovascular dysfunction	Prenatal phthalates → ↑ DNA methylation changes (ECE1, SCNN1G) → ↑ blood pressure in offspring
[82]	Urinary DEHP metabolites quantified via LC–MS/MS Global DNA methylation measured in blood samples (ELISA-based 5-methylcytosine quantification) Carotid intima–media thickness (CIMT) assessed by ultrasound imaging Statistical correlation and regression analyses	Human study: Young individuals, Taiwanese Treatment: No experimental treatment; cross-sectional observational study	Phthalate exposure is associated with epigenetic alterations that may contribute to early atherosclerotic changes	MEHP → ↑ global DNA methylation → ↑ carotid intima–media thickness
[117]	Urinary MEHP quantification: LC–MS/MS Serum apoptotic microparticles (CD31+/CD42a−, CD14+) measured by flow cytometry Global DNA methylation: ELISA-based 5-methylcytosine quantification Mediation analysis linking MEHP, DNA methylation, and microparticle levels	Human study: Young individuals, Taiwanese Treatment: No experimental treatment; cross-sectional observational study	Phthalate exposure induces vascular cell apoptosis via epigenetic modulation, linking DNA methylation changes to subclinical vascular injury	MEHP → ↑ global DNA methylation → ↑ apoptotic microparticles (CD31+/CD42a−, CD14+)
[118]	Urinary phthalate metabolites quantified via LC–MS/MS Plasma miRNAs (miR-146a and others) quantified by qRT-PCR Arterial stiffness measured via pulse wave velocity (PWV) Statistical mediation analysis linking phthalates, miRNAs, and PWV	Human study: Adults Treatment: No experimental treatment; panel observational study	Phthalate exposure modulates cardiovascular-related miRNAs, promoting subclinical vascular dysfunction linked to IHD risk	MMP/MBP → ↑ miR-146a → ↑ arterial stiffness
[35]	Urinary MEHP quantified via LC–MS/MS Plasma/serum miRNAs (miR-155, miR-208a) measured by qRT-PCR Statistical comparison between CHD patients and controls	Human study: CHD patients vs healthy controls Treatment: No experimental treatment; case-control study)	Phthalate exposure modulates cardiovascular-related miRNAs, promoting atherogenesis and CHD risk	MEHP → ↑ miR-155 / ↑ miR-208a → ↑ vascular inflammation /endothelial dysfunction
**Lipid Accumulation**				
**References**	**Methods**	**Models and Treatments**	**Biological Meaning**	**Identified/Described Mechanisms**
[119]	Lipid droplet visualization: Oil Red O staining, high-content cellomics imaging Gene/protein expression: qRT-PCR & Western blot for C/EBPα, PPARγ, downstream targets Metabolomics: LC–MS-based profiling; pathway analysis (glyceroneogenesis, fatty acid synthesis)	In vitro model: 3T3-L1 preadipocytes Treatment: BBP, 0.1–100 μM; 24–96 h	Phthalate promotes preadipocyte differentiation and metabolic reprogramming, leading to enhanced lipid storage	BBP → ↑ C/EBPα & ↑ PPARγ → ↑ adipogenesis/lipid accumulation
[120]	Lipid accumulation: Oil Red O staining miRNA expression: qRT-PCR for miR-34a-5p Adipogenic markers (C/EBPα, PPARγ) assessed by Western blot and qRT-PCR	In vitro model: 3T3-L1 preadipocytes Treatment: BBP, 1–50 μM; 48, 96 h	Phthalate promotes preadipocyte differentiation through miRNA-mediated signaling, enhancing lipid storage even without exogenous adipogenic stimuli	BBP → ↑ miR-34a-5p → ↑ adipogenesis/lipid accumulation
[121]	Gene/protein expression: qRT-PCR and Western blot for PPARγ and Fabp4 Lipid accumulation: Oil Red O staining Cardiomyocyte differentiation monitored via immunostaining for cardiac markers	In vitro model: P19 embryonal carcinoma cells differentiating into cardiomyocytes Treatment: DEHP, 10–100 μM; 7 days	Phthalate exposure promotes metabolic reprogramming and lipid storage during cardiomyogenic differentiation	DEHP → ↑ PPARγ → ↑ Fabp4 → ↑ lipid accumulation
[122]	Monocyte adhesion assay: Fluorescently labeled monocytes co-cultured with endothelial cells Gene/protein expression: qRT-PCR & Western blot for adhesion molecules and chemokines Histological analysis in mice: Immunostaining of vascular tissue for adhesion molecules	In vitro model: Human umbilical vein endothelial cells (HUVECs) Treatment: DBP, 1–50 μM; 24, 48 h Animal model: Male C57BL/6 mice Treatment: DBP, 50 mg/kg/day, oral gavage; 7 days	Phthalate promotes endothelial activation, facilitating initiation and progression of atherosclerosis	DBP → ↑ adhesion molecules/↑ chemokines → ↑ monocyte adhesion
[113]	Foam cell formation: Oil Red O staining, microscopy Lipid uptake: Fluorescently labeled oxLDL assay Lipid profile in mice: Serum cholesterol, triglycerides, LDL/HDL measurement Histology: Aortic plaque assessment via Oil Red O and immunostaining	In vitro model: Human THP-1 macrophages Treatment: DEHP, 10–50 μM; 24–72 h Animal model: ApoE-deficient mice, male, 8 weeks Treatment: DEHP, 50 mg/kg/day, oral gavage; 12 weeks	Phthalate promotes macrophage lipid accumulation and accelerates atherosclerotic plaque development	DEHP → ↑ oxLDL uptake → ↑ foam cell formation
[123]	Gene expression: qRT-PCR for PXR target genes (lipogenesis, ceramide synthesis) Protein expression: Western blot for PXR and downstream targets Lipid profiling: Serum and hepatic lipid quantification Histology: Liver tissue staining for lipid accumulation	Animal model: C57BL/6 mice, male Treatment: DCHP, 50 mg/kg/day, oral gavage; 4 weeks	Phthalate promotes lipid synthesis and alters lipid homeostasis via nuclear receptor signaling	DCHP → ↑ PXR activation → ↑ lipogenic & ceramide genes
[124]	Anthropometric measurements: BMI, waist circumference Blood biochemistry: LDL-C, triglycerides quantification Statistical analysis: Correlation between urinary phthalate metabolite levels and cardiometabolic markers	Human cohort: Children and adolescents Treatment: No experimental treatment; observational study	Phthalate exposure associated with increased cardiometabolic risk and lipid accumulation in pediatric population	Phthalate metabolites (MBP, MBzP, MEHP, MMP, MEOHP) → ↑ BMI/↑ waist circumference/↑ LDL-C/↑ triglycerides
[125]	Anthropometric measures: Waist circumference, BMI Blood biochemistry: Triglycerides, fasting glucose, lipid profile Statistical analysis: Association between urinary phthalate metabolites and prevalence of metabolic syndrome	Human cohort: 25,365 individuals across nine cross-sectional observational studies Treatment: No experimental treatment; observational	Phthalate exposure linked to dysregulated lipid homeostasis, central obesity, insulin resistance, ↑ triglycerides; established risk factors for atherosclerosis and IHD	Phthalate metabolites (MEHP, MBzP, MiBP, MMP) → ↑ risk of metabolic syndrome
[126]	Maternal urinary phthalate metabolite quantification (DEP, DBP, DEHP) Child anthropometrics: BMI, weight-for-age, height-for-age Statistical analysis: Association between maternal phthalate levels and child BMI/obesity outcomes	Human cohort: Mother–child pairs Treatment: No experimental treatment; prospective observational study	Prenatal phthalate exposure linked to metabolic disorders in offspring, promoting higher adiposity and obesity risk	Maternal urinary phthalate metabolites (DEP, DBP, DEHP) → ↑ BMI/↑ risk of childhood overweight/obesity

Abbreviations: ↓: decrease of; ↑: increase of; 8-OHdG: 8-hydroxy-2-deoxyguanosine; ΔΨm: mitochondrial membrane potential; ACSL4: acyl-CoA synthetase long-chain family member 4; Akt1: protein kinase B; alpha; ApoE^−^/^−^: apolipoprotein E-deficient; ASC: apoptosis-associated speck-like protein containing a CARD; ATP: adenosine triphosphate; BBP: benzyl butyl phthalate; Bcl-2: B-cell lymphoma 2; BMI: body mass index; CAD: coronary artery disease; CCK-8: cell counting kit-8; CD14: cluster of differentiation 14; CD31: cluster of differentiation 31; CD42a: platelet glycoprotein Ia; cGAS: cyclic GMP-AMP synthase; CHD: coronary heart disease; CIMT: carotid intima–media thickness; CK-MB: creatine kinase–MB isoenzyme; COX-2: cyclooxygenase-2; CRP: C-reactive protein; CVD: cardiovascular disease; DBP: dibutyl phthalate; DCFH-DA: 2′,7′-dichlorodihydrofluorescein diacetate; DCHP: dicyclohexyl phthalate; DEG: differentially expressed gene; DEHP: di-(2-ethylhexyl) phthalate; DEP: diethyl phthalate; DMP: dimethyl phthalate; DMR: differentially methylated region; ΔΨm: mitochondrial membrane potential; EA.hy926: human vascular endothelial cell line; ECE1: endothelin converting enzyme 1; ELISA: enzyme-linked immunosorbent assay; Fabp4: fatty acid binding protein 4; FasL: Fas ligand; GAS5: growth arrest-specific 5; C/EBPα: CCAAT/enhancer-binding protein alpha; GFP-LC3: green fluorescent protein–microtubule-associated protein 1 light chain 3; GPX4: glutathione peroxidase 4; GSDMD: gasdermin D; GSH: glutathione; HDL-C: high-density lipoprotein cholesterol; HF: heart failure; HO-1: heme oxygenase-1; hs-CRP: high-sensitivity C-reactive protein; HUVEC: human umbilical vein endothelial cells; ICAM-1: intercellular adhesion molecule 1; IHD: ischemic heart disease; IL-1β: interleukin-1 beta; IL-18: interleukin-18; JAK-STAT: Janus kinase–signal transducer and activator of transcription; JC-1: 5,5′,6,6′-tetrachloro-1,1′,3,3′-tetraethylbenzimidazolylcarbocyanine iodide; LC3: microtubule-associated protein 1 light chain 3; LC-MS/MS: liquid chromatography–tandem mass spectrometry; LDH: lactate dehydrogenase; LDL-C: low-density lipoprotein cholesterol; lncRNA: long non-coding RNA; M1: classically activated macrophages; MBP: monobutyl phthalate; MBzP: monobenzyl phthalate; MCP-1: monocyte chemoattractant protein-1; MDA: malondialdehyde; MEcPP: mono-(2-ethyl-5-carboxypentyl) phthalate; MEHP: mono-(2-ethylhexyl) phthalate; MEOHP: mono-(2-ethyl-5-oxohexyl) phthalate; MEP: monoethyl phthalate; MMP: mono-methyl phthalate; MiBP: mono-isobutyl phthalate; miR/miRNA: microRNA; MYOCD: myocardin; mtDNA: mitochondrial DNA; MTT: 3-(4,5-dimethylthiazol-2-yl)-2,5-diphenyltetrazolium bromide; NHANES: National Health and Nutrition Examination Survey; NLRP3: NOD-like receptor family pyrin domain containing 3; NO: nitric oxide; Nrf2: nuclear factor erythroid 2–related factor 2; oxLDL: oxidized low-density lipoprotein; PAI-1: plasminogen activator inhibitor-1; PI: propidium iodide; PI3K: phosphoinositide 3-kinase; PIK3CA: phosphatidylinositol 4,5-bisphosphate 3-kinase catalytic subunit alpha; PPARγ: peroxisome proliferator-activated receptor gamma; PTGS2: prostaglandin-endoperoxide synthase 2; PWV: pulse wave velocity; PXR: pregnane X receptor; qRT-PCR: quantitative reverse transcription polymerase chain reaction; RMSD: root mean square deviation; RMSF: root mean square fluctuation; ROS: reactive oxygen species; RR: risk ratio; RRBS: reduced representation bisulfite sequencing; SCNN1G: sodium channel epithelial 1 gamma subunit; SPHK1: sphingosine kinase 1; S1PR2: sphingosine-1-phosphate receptor 2; SOD: superoxide dismutase; SP1: specificity protein 1; STING: stimulator of interferon genes; TBARS: thiobarbituric acid reactive substances; TEM: transmission electron microscopy; TF: tissue factor; TGF-β1: transforming growth factor beta; THP-1: human monocytic cell line; VCAM-1: vascular cell adhesion molecule 1; VSMC: vascular smooth muscle cell.

Animal studies have shown that phthalate exposure is associated with upregulation of inflammatory mediators, further exacerbating cardiovascular injury and promoting conditions such as atherosclerosis [127]. In mice, DEHP exposure markedly increases cardiac oxidative stress markers including MDA and protein carbonyl levels, indicating ROS overproduction and lipid peroxidation, and also disrupts lipid homeostasis by elevating total cholesterol, triglycerides, and LDL cholesterol, while reducing HDL cholesterol, ultimately raising the atherogenic index and atherosclerosis risk [98]. Disturbances in cholesterol homeostasis, along with inflammation in endothelial cells, were previously observed in a mouse model chronically treated with DEHP [99]. Furthermore, DEHP exposure has also been shown to cause myocardial injury in terms of disorganized fibers, collagen deposition, and elevated serum markers of cardiac damage (creatine kinase MB) and inflammation (CRP). Mechanistically, DEHP and its metabolite MEHP activate the SPHK1/S1PR2 sphingolipid pathway, which triggers cardiomyocyte pyroptosis and subsequent cell death [100].

Transcriptomic and flow cytometry analyses in rats exposed to DMP revealed ROS-mediated activation of both intrinsic (Bcl-2 family, Bax/Bcl-2 ratio) and extrinsic (Fas/FasL pathway) apoptotic cascades, culminating in caspase-dependent cardiomyocyte apoptosis [101]. Phthalates also promoted the upregulation of pro-inflammatory cytokines and activation of the NLRP3 inflammasome, leading to increased interleukin (IL)-1β and IL-18 production, as well as enhanced neutrophil and macrophage infiltration in cardiac tissue after myocardial injury [102]. Murine models exposed to DEHP further exhibited oxidative stress, as indicated by increased lipid peroxidation and mitochondrial damage, alongside activation of the Nrf2/heme oxygenase-1 pathway and induction of ferroptosis in cardiomyocytes [103]. Finally, exposure to DBP, particularly when combined with a high-fat diet, exacerbated cardiac fibrosis and dysfunction in rats by inducing oxidative stress, characterized by elevated ROS and lipid peroxidation (MDA) and depletion of antioxidant defenses (GSH). This redox imbalance led to membrane damage, mitochondrial dysfunction, and activation of inflammatory and pyroptotic pathways (upregulation of NLRP3 inflammasome, caspase-1, and gasdemin D), while also disrupting lipid metabolism and endocrine homeostasis, ultimately promoting structural myocardial damage and cardiotoxicity relevant to CVD and IHD pathogenesis [104]. DEHP also promotes vascular inflammation by inducing tissue factor-bearing microparticle release from human M1 macrophages via activation of the TGF-β1/Smad/plasminogen activator inhibitor-1 (PAI-1) signaling pathway. This mechanism increases tissue factor expression and the formation of procoagulant microparticles, thereby linking DEHP exposure to inflammatory and thrombotic processes that contribute to the development and progression of cardiovascular disease [105].

In a human cohort of more than 1000 individuals, urinary concentrations of several phthalate metabolites (MEP, MBP, MiBP, MBzP, and the sum of six phthalate metabolites) were positively correlated with higher blood pressure and increased prevalence of hypertension, attributing the higher cardiovascular risk to the phthalate-induced oxidative stress with elevated levels of oxidative stress biomarkers, specifically 8-hydroxy-2-deoxyguanosine and malondialdehyde (MDA) [106]. Elevated MDA together with reduced SOD activity were also observed in association with higher urinary MEHP levels in a previous case-control study enrolling CHD patients [35], supporting the link between this phthalate-induced oxidative stress and cardiovascular pathology. In a younger population, a positive association was also observed between MEHP levels and the presence of endothelial and platelet microparticles, which are generated during cell death and inflammation processes involved in atherosclerosis development. Urinary concentrations of DEHP metabolites were compared to serum markers indicative of endothelial apoptosis (CD31^+^/CD42a^−^), platelet apoptosis (CD31^+^/CD42a^+^), and activation of monocytes, macrophages, and neutrophils (CD14) [75].

Moreover, CHD patients with elevated urinary levels of DEHP metabolites exhibited higher levels of atherothrombotic and inflammatory markers (high-sensitivity CRP-hs-CRP, fibrinogen, and D-dimer), supporting a mechanistic link between phthalate exposure, vascular inflammation, and thrombosis [88]. Oxidative stress, inflammation, and apoptosis at the cardiovascular level were indicated as molecular mechanisms involved in phthalates-induced CVD from a network toxicology analysis with pathway enrichment and protein–protein interaction that identified phosphatidylinositol 3′-kinase (PI3K)-Akt and JAK-STAT signaling (inflammation and cell survival), BCL2 (apoptosis regulation), and PIK3 catalytic subunit (inflammatory and survival signaling) as central targets [33]. Molecular docking analyses confirmed strong binding affinities between phthalate metabolites and these proteins, reinforcing the plausibility that phthalate exposure contributes to cardiovascular toxicity by promoting oxidative stress, inflammatory signaling, and apoptosis in cardiac and vascular cells [33].

### 4.2. Mitochondrial DNA Alterations and Dysfunction

Mitochondrial DNA (mtDNA) is a small, circular, double-stranded genome (~16.6 kb in humans) encoding 13 essential subunits of the oxidative phosphorylation (OXPHOS) system, as well as 22 tRNAs and two rRNAs required for intramitochondrial protein synthesis [128]. Each mitochondrion contains multiple copies of mtDNA, and each cell harbors hundreds to thousands of mtDNA copies, depending on its energetic demand. mtDNA copy number reflects the quantity of mitochondrial genomes per cell and can be used to indirectly measure mitochondrial function. Alteration in mtDNA copy number is closely associated with several diseases, including CVD [109]. Additionally, mtDNA copy number is currently regarded as an emerging biomarker of environmental exposure [129].

Cardiomyocytes, due to their exceptionally high requirement for ATP to sustain continuous contractile activity, are particularly enriched in mitochondria and mtDNA, making mitochondrial integrity critical for normal cardiac function [130,131]. Unlike nuclear DNA, mtDNA lacks protective histones and has limited DNA repair capacity, making it highly susceptible to damage induced by ROS, environmental toxicants, and metabolic stress. mtDNA injury can manifest as point mutations, large-scale deletions, strand breaks, or epigenetic alterations, ultimately compromising mitochondrial biogenesis and respiratory capacity [132,133]. Such damage directly compromised oxidative phosphorylation by impairing the expression and function of mitochondrially encoded subunits of the respiratory chain, leading to reduced electron transport efficiency and defective ATP production [134,135]. Consequently, dysfunctional mitochondria exhibit increased electron leakage and excessive generation of ROS, which further amplifies mtDNA damage and establishes a self-perpetuating cycle of mitochondrial oxidative stress [135,136].

It is well established that mitochondrial dysfunction is strongly correlated with the development and progression of CVD and IHD [134]. In vascular endothelial cells, this redox imbalance promotes endothelial dysfunction through reduced nitric oxide bioavailability, increased inflammation, and impaired vasodilatory capacity, key early events in atherogenesis [134]. In cardiomyocytes, chronic mitochondrial dysfunction and ATP depletion drive maladaptive myocardial remodeling, characterized by altered calcium handling, fibrosis, and progressive contractile dysfunction [134,136].

At the molecular level, phthalates interfere with mitochondrial redox homeostasis by increasing ROS production and impairing antioxidant defense systems, thereby exacerbating oxidative damage to mitochondrial membranes, proteins, and mtDNA. In detail, exposure to DEHP has been shown to disrupt mitochondrial dynamics and biogenesis, leading to defective oxidative phosphorylation and further reductions in mtDNA copy number [30,34], a surrogate marker of mitochondrial biogenesis and function. In vitro studies using zebrafish-derived cells showed that DBP exposure induces mitochondrial fragmentation, loss of mitochondrial membrane potential, and impaired ATP synthesis, reflecting a collapse of mitochondrial bioenergetic capacity [107].

Coherently, DBP during early developmental stages of zebrafish models induced mtDNA stress responses, characterized by increased mtDNA lesions, reduced mtDNA copy number, and activation of mitochondrial damage signaling pathways. Additionally, DBP-induced mtDNA damage triggers the release of mtDNA into the cytosol, activating the cGAS-STING inflammatory signaling pathway and further linking mitochondrial injury to cellular inflammation [108]. These mitochondrial perturbations were shown to activate downstream cell-death pathways, including intrinsic apoptosis and inflammatory signaling cascades, thereby connecting phthalate-induced mitochondrial dysfunction to tissue injury [107,108].

A recent population-based studies demonstrated that reduced mtDNA copy number was strongly associated with increased risk of atherosclerosis and major cardiovascular events, supporting the central role for mtDNA integrity in CVD pathogenesis, as proved by the reduced leukocyte mtDNA copy number associated with a higher risk of incident CHD [109]. Moreover, a recent systematic review and meta-analysis encompassing multiple observational studies confirmed that lower mtDNA copy number was significantly associated with major risk for CVD (summary Relative Risk—RR 2.09, 95% CI 1.59–2.75), coronary heart disease (RR 1.70, 95% CI 1.29–2.24), heart failure (RR 1.43, 95% CI 1.15–1.79), stroke (RR 1.88, 95% CI 1.08–3.28), and all-cause mortality (RR 1.33, 95% CI 1.21–1.47), supporting its value as a robust biomarker of mitochondrial dysfunction and adverse cardiovascular outcomes [110].

Collectively, the convergence of oxidative stress, mtDNA damage, and bioenergetic failure provides a molecular framework through which phthalate exposure may contribute to cardiovascular pathology, highlighting environmental toxicants as critical and underappreciated determinants of cardiovascular disease risk, including IHD [33,34].

### 4.3. DNA Methylation and Non-Coding RNAs

DNA methylation is an epigenetic modification that refers to the covalent addition of a methyl group to the cytosine base within CpG dinucleotides, typically leading to transcriptional repression by restricting access of transcription factors and recruiting methyl-binding proteins that promote a closed chromatin conformation, thereby reducing gene accessibility and silencing gene expression [137].

Another major epigenetic mechanism involves non-coding RNAs, particularly microRNAs (miRNA) and long non-coding RNA (lncRNA). These molecules mainly exert post-transcriptional control by promoting mRNA degradation or inhibiting translation, but they can also interact with chromatin-modifying complexes to affect transcriptional regulation [138]. These epigenetic processes are responsive to both internal and external determinants, including environmental pollutants, and are recognized as key contributors to epigenetic reprogramming and heightened CAD risk [139].

In vitro evidence indicates that DBP accelerates atherosclerosis through two distinct miRNA-mediated mechanisms. First, DBP exposure upregulates miR-139-5p, which directly targets and suppresses myocardin, a central transcriptional regulator of the vascular smooth muscle cell (VSMC) contractile phenotype. This suppression promotes the phenotypic switch of VSMCs from a contractile to a synthetic state, a process that contributes to plaque formation and instability in atherosclerosis [111]. Second, DBP downregulates miR-137-3p, which normally inhibits specificity protein 1 (SP1). Reduced miR-137-3p leads to increased SP1 expression, which in turn upregulates monocyte chemoattractant protein-1 (MCP-1). Elevated MCP-1 enhances monocyte recruitment to the endothelium, a critical early event in atherogenesis [112].

Moreover, DEHP has also been shown to provoke epigenetic changes that promote atherogenesis. Liu et al. demonstrated that DEHP exposure increases expression of GAS5, a lncRNA that sequesters miR-145-5p, leading to pathological proliferation and enhanced apoptosis in a VSMC model, processes that may contribute to vascular damage and plaque instability and, ultimately, CAD progression [113]. In the same study, GAS5 knockdown reduced foam-cell formation from macrophages, supporting the role of this lncRNA in atherogenesis.

In mice, the effect of DEHP on DNA methylation status was shown to be significant even during the perinatal period. Indeed, exposure to this phthalate results in the presence of thousands of differentially methylated regions and hundreds of differentially expressed genes that persistently alter cardiac cell-type proportion and composition, influencing cardiovascular risk later in life [114]. These results are consistent with the earlier observations of Svoboda et al. [115].

Worrying effects related to phthalate exposure on cardiovascular risk in offspring have also been documented in humans. A prospective cohort study found that higher urinary concentrations of phthalate metabolites (MMP, MEP and MEcPP) during the third trimester of pregnancy of 198 women were associated with elevated blood pressure of their children later when they were in preschool-age, and this relationship is mediated by DNA methylation changes in genes (e.g., *ECE1*, *SCNN1G*) related to hypertension risk [116].

Lin et al. demonstrated a positive correlation between urinary MEHP concentration in a group of young individuals, and carotid intima–media thickness, a marker of subclinical atherosclerosis, and increased global DNA methylation [82]. In the same cohort, elevated levels of apoptotic microparticles derived from vascular cells (CD31/CD42a and CD14) were also observed, suggesting that phthalate exposure may promote vascular cell apoptosis through global DNA methylation changes acting as mediators in this pathway [117]. Furthermore, several human studies indicate that phthalates can influence levels of miRNAs implicated in cardiovascular dysfunction and IHD. A panel study reported that several phthalate metabolites, in particular MMP and MBP, were associated with increased levels of some miRNAs, including miR-146a, which is linked to arterial stiffness [118], a marker and a mediator of cardiovascular risk, closely associated with atherosclerotic burden [140]. In a case-control study of CHD patients, urinary MEHP concentration correlated with higher levels of miR-155 and miR-208a, implicated in vascular inflammation, endothelial dysfunction, and atherogenesis, in comparison to healthy controls [35].

### 4.4. Lipid Accumulation

Lipid accumulation is a fundamental pathological process underlying the development of IHD. The progressive retention of atherogenic lipoproteins within the arterial wall initiates and sustains chronic vascular inflammation, ultimately driving atherosclerotic plaque formation and progression. Elevated circulating lipid levels, particularly LDL cholesterol, are strongly associated with plaque burden and represent a major, cumulative determinant of cardiovascular risk across the lifespan. Lipid accumulation actively promotes endothelial dysfunction, oxidative stress, VSMC proliferation, and plaque instability, thereby increasing susceptibility to acute ischemic events such as MI [141].

Evidence from the literature describes phthalates’ ability to trigger lipid accumulation and related metabolic processes. BBP promotes adipogenesis in 3T3-L1 preadipocytes by increasing both the number and size of lipid droplets in a dose-dependent manner, through the upregulation of key adipogenic transcription factors, including C/EBPα and PPARγ, as well as their downstream target genes and proteins, indicating activation of the adipogenic differentiation pathway. Metabolomic analysis revealed that BBP alters metabolic profiles by enhancing pathways involved in gluconeogenesis and fatty acid synthesis, both central to lipid accumulation [119]. The capability of BBP to enhance adipocyte differentiation was subsequently confirmed by Meruvu et al., who attributed this effect to elevated expression of the miRNA-34a-5p signaling pathway [120]. Similarly, DEHP exposure activates PPARγ and upregulates its downstream target fatty acid binding protein 4, promoting pathways involved in lipid accumulation in cardiomyogenic-differentiating P19 embryonal carcinoma cells [121].

Moreover, phthalates can facilitate initiation and progression of atherosclerotic plaque. DBP exposure upregulates cellular adhesion molecules and chemokines, leading to a marked increase in monocyte adhesion to the endothelium both in vitro and in vivo [122]. In macrophages, DEHP exposure enhances oxidized LDL uptake and foam-cell formation, accelerating plaque development. Consistently, DEHP administration in ApoE-deficient mice leads to altered lipid profiles and a marked acceleration of atherosclerotic plaque progression [113].

Another animal study shed light on an interesting effect exerted by which DCHP may contribute to lipid dysregulation: it acts as a potent and selective agonist of the intestinal pregnane X receptor, leading to its activation, thereby upregulating genes involved in lipogenesis and ceramide synthesis [123].

A remarkable number of human studies, discussed in two systematic reviews and meta-analyses, highlight the correlation between phthalate exposure and conditions attributable to lipid accumulation and promoting IHD. In the first meta-analysis, Golestanzadeh et al. found that MBP, MBzP, MEHP, MMP, and MEOHP were associated with lipid accumulation in children and adolescents, as evidenced by significant correlations with increased BMI, waist circumference, LDL cholesterol, and triglyceride levels [124]. In the second, Mérida et al. integrated data from nine cross-sectional observational studies involving 25,365 individuals, linking LMW and HMW phthalates and their metabolites (such as MEHP, MBzP, MiBP, and MMP) to higher odds of metabolic syndrome, a condition characterized by dysregulated lipid homeostasis, central obesity, insulin resistance, and elevated triglycerides, all established risk factors for atherosclerosis and IHD [125]. Furthermore, prenatal exposure to phthalates was shown to cause metabolic disorders in offspring. Indeed, maternal urinary concentrations of DEP, DBP, and DEHP metabolites were associated with higher BMI levels and a greater likelihood of childhood overweight or obesity [126], supporting the role of phthalates in cardiovascular risk.

**Figure 2:**
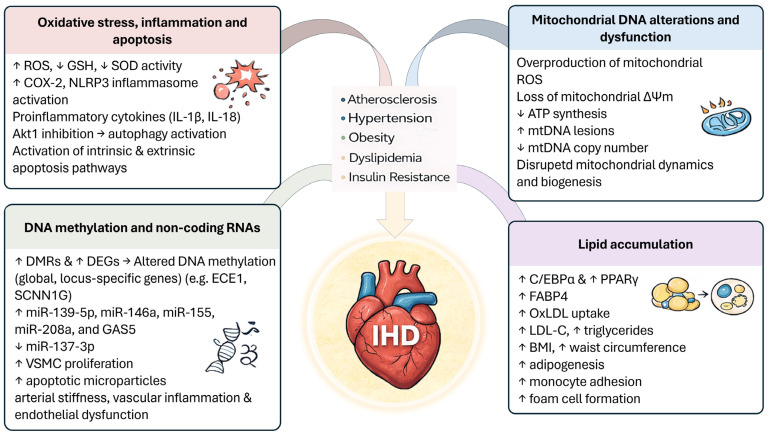
Phthalate-induced mechanisms related to ischemic heart disease. Image partly generated with AI Microsoft Copilot 365. Abbreviations: Akt1: protein kinase B; ATP: adenosine triphosphate; BMI: body mass index; C/EBPα: CCAAT/enhancer-binding protein alpha; COX-2: cyclooxygenase-2; DEGs: differentially expressed genes; DMRs: differentially methylated regions; DNA: deoxyribonucleic acid; ΔΨm: mitochondrial membrane potential; ECE1: endothelin converting enzyme 1; FABP4: fatty acid-binding protein 4; GSH: glutathione; IHD: ischemic heart disease; IL: interleukin; LDL-C: low-density lipoprotein cholesterol; miR: microRNA; mtDNA: mitochondrial DNA; NLRP3: NOD-, LRR- and pyrin domain-containing protein 3; OxLDL: oxidized low-density lipoprotein; PARPγ: peroxisome proliferator-activated receptor gamma; ROS: reactive oxygen species; SCNN1G: sodium channel epithelial 1 subunit gamma; SOD: superoxide dismutase; VSMC: vascular smooth muscle cell.

Figure 2 summarizes cellular and molecular mechanisms underlying phthalate-induced ischemic heart disease.

## 5. The Present and Future of Research: Intelligent Approaches in Studying Phthalate Exposure and Cardiovascular Risk

Recently, research on environmental health and cardiovascular diseases has benefitted from the growing integration of artificial intelligence (AI) models, especially those of machine learning (ML), to identify relationships between exposures, cardiometabolic pathways and clinical outcomes. Traditional statistics have been used to investigate such relationships in the past, failing however at retrieving such relationships in case of non-linear, complex associations. Therefore, the adoption of AI, novel in the field, enables improving the understanding around the mechanisms of how phthalate exposure can contribute to CVD and IHD.

### 5.1. AI for Detecting and Modeling Phthalate Exposure and Health Outcomes

AI analytics has been recently applied more often to model the health effects of phthalates. Among them, a large-scale study was conducted using demographic and laboratory data from the Taiwan Biobank [142]. In this regard, the research developed a prediction model devoted to the examination of the relationship between phthalate esters (particularly DEHP), lifestyle factors, and disease outcomes. The model assessed the risk of developing certain diseases using several AI-based algorithms, including logistic regression, artificial neural networks, and Bayesian networks. According to the results obtained, phthalate esters showed a greater impact on bone and joint problems than on cardiovascular conditions. Furthermore, DEHP metabolites, such as mono(2-carboxymethylhexyl) phthalate, MnBP, and MEP, appear to leave higher residue in females than in males, with statistically significant differences. MEP levels were also found to be lower in individuals who exercised regularly than those who did not, demonstrating that phthalate-related risk patterns differ by sex and physical activity levels [142].

Another key application of AI in this regard, even if not directly related to cardiovascular health, is the enhancement of environmental detection technologies. In particular, ML was embedded into an electrochemical aptasensing platform aimed at reliably analyzing DEHP concentrations in water at the upper, mid, and lower layers of three sites across South Korean rivers [143]. In fact, researchers failed to find satisfying solutions when solely relying on sensor application due to signal drift, biofouling, and limited specificity, especially with pH fluctuations. In that study, an ML-powered platform, using a Conventional Generative Adversarial Network (cGAN) model for data augmentation, useful in imbalanced datasets and working well for images, audio, text embeddings and time-series, and a hybrid Phthalate Boosting (PLBoost) algorithm for a robust multi-layer concentration analysis, significantly improved the DEHP prediction accuracy (97.11%) compared to those of the liquid–liquid extraction/gas chromatography/mass spectrometry (LLE-GC–MS) measurement [143].

### 5.2. AI in Cardiovascular Risk Prediction and IHD

In the last years, AI has quickly emerged to transform the paradigm of cardiovascular risk prediction in general and specifically when applied to IHD through the integration of multimodal, multiparametric data, due to its enhanced capacity for modeling. In this regard, a literature review was recently published highlighting the role of AI in creating potentially useful models to improve IHD prevention, relying on multimodal inputs including biomarkers, clinical records, data captured by wearable tools, and imaging [144]. Such models outperformed classical risk scores in terms of predictive accuracy, further supporting their use in personalization of preventive strategies.

More specifically, a Gradient Boosting Machine (GBM) model, trained on Electronic Health Record (EHR)-derived data from more than 250,000 patients, outperformed traditional methods for atherosclerotic risk prediction (AUC: 0.835 vs. 0.775), and improved accuracy of other ML models thanks to its capability of handling missing data and of working on mixed types, different from the typical lab use cases [145]. A set of ML models was also used to identify secondary CVD in a cohort of more than 32,000 patients, with the XGBoost, specifically optimized for structured, tabular data, performing best, with an AUC of 0.70 for all cases and 0.71 for those with atherosclerosis CVD, outperforming other ML models. Also, thanks to ML approach, some additional risk factors for adverse events were identified, including education level and primary care visits [146]. However, such results must be considered cautiously due to the lack of standardization of some approaches and of validation in real-world test benchmarks.

In terms of the discovery of biomarkers for CVD, ML can identify genetic variants of single nucleotide polymorphisms potentially overlooked by most traditional statistics. A Feature Selection algorithm, based on ML, can identify panels with 50 genetic markers to achieve 80% accuracy when combined with known risk factors [147]. In the same field, XGBoost was used to develop a protein-based risk model in the SMART derivation cohort and was validated in the Athero-Express cohort, outperforming (AUC: 0.80 vs. 0.77) the classical clinical risk model, with good calibration, too [148]. Lipidomics was also approached to draft a risk score within a ML workflow using ridge regression on the AusDiab study (n  =  10,339) and validated within the Busselton Health Study (n  =  4492), with a noteworthy improvement in CVD risk prediction, highlighting the utility of blood biomarkers to improve the IHD risk stratification beyond traditional tools [149].

Another emerging area in which AI shows considerable promise is the analysis of patient-generated data, thanks to the fast advancements in remote sensing technologies and remote patient monitoring. This new technological approach guarantees the availability of large amounts of data, ideal for the implementation of AI models. Nevertheless, its implementation in the field of IHD is still in its infancy and far from being demonstrated in terms of efficacy and advantages. A randomized control trial tried to understand the role of variables integrated within a smart device on blood pressure control in patients having suffered from a previous MI, with good results in terms of tolerability, yet without a significant improvement in the control of blood pressure [150]. Fitbit-derived data, together with psychometric analysis, were used to predict cardiovascular risk in those with stable ischemic heart disease, with an association retrieved between data acquired and N-terminal pro-brain natriuretic peptide, as well as between biometric data and cardiac-specific troponin-I [151].

AI has also begun to be applied in the field of cardiac signal processing and diagnostic imaging. The DL model SEER was developed using a large dataset of 12-lead ECG recordings and was capable of predict 5-year cardiovascular mortality with an AUC of 0.83, and atherosclerotic disease with an AUC of 0.67, with further enhancement of performances when combined with the Pooled Cohort Equations [152]. In fact, SEER demonstrated here its ability to capture subtle, non-linear electrical signatures associated with long-term cardiovascular risk. Furthermore, an AI-based tool developed starting from data from 7 million patients was able to identify cardiovascular conditions with an AUROC between 0.85 and 0.94, depending on the specific analysis to be carried out [153].

When it comes to imaging, several studies have validated AI approaches for calculating significant features from Coronary Artery Calcium (CAC) scans, in particular with Convolutional Neural Network (CNN) models, outperforming classical scores, like the Agatston, in predicting coronary heart disease in different follow-up periods [154]. Radiomics, a relatively novel method for automated feature extraction from imaging, is also increasingly applied in cardiovascular imaging in an increased fashion. Notably, an analysis of radiomic features from CAC on CT in 624 patients from the Framingham Heart Study demonstrated improved prediction and enhanced discriminatory ability compared with the benchmark analysis carried out previously [155].

ML is also adopted in combination with CT to achieve high accuracy in detecting stenosis and improving decision-making by reducing unnecessary interventions and predicting long-term outcomes [156]. In fact, AI has demonstrated an excellent capability to improve plaque identification and evaluation on a cohort of 232 patients following a CNN approach, supporting the classical Coronary Computed Tomography Angiography (CCTA) analysis [157]. All those results were made possible thanks to the matching between specific characteristics of imaging techniques features that match well with the core strengths of convolutional architectures like CNNs. Nevertheless, it should be noted that current international prevention guidelines do not recommend CCTA as a screening strategy in guiding primary prevention therapy, although further clinical trials employing advanced AI models may support the eventual integration of this modality into clinical guidelines [158].

AI has also proven valuable in the analysis of stress echocardiograms, achieving 92.7% specificity and 84.4% sensitivity in identifying severe coronary artery disease [159], while also enhancing clinical interpretation [160].

Recently, DL models have demonstrated their performance as superior to conventional models, in large multicenter registries like the REFINE–SPECT registry, featuring more than 20,000 participants, tailored at the automated analysis of nuclear myocardial perfusion imaging to predict CAD and cardiovascular outcomes [161].

Finally, a comprehensive state-of-the-art overview published in the European Heart Journal underscored the substantial potential of AI to promote cardiovascular health at the population level. The authors highlighted a wide range of applications—from early detection of cardiovascular events and continuous monitoring of disease progression to the optimization of preventive, personalized interventions. The review also emphasized the value of integrating large-scale registry and biobank data within AI frameworks, noting that such approaches could be extended to incorporate additional data domains, including environmental exposures such as phthalates [162].

### 5.3. Phthalates and Cardiometabolic Pathways: AI Role in Their Integration

Beyond being used in the typical demands of risk prediction, AI approaches are being applied continuously to the aim of multimodal modeling in the cardiometabolic field. In this domain, a recent review, published in *Cell Metabolism*, highlighted how transformer-based and multimodal DL systems can integrate different, broad datasets, which include genetic information, metabolic and inflammatory biomarkers, sleep indicators, and behavioral determinants, to jointly model mechanisms, which are at the basis of cardiometabolic disease. All in all, the models described are capable of being adapted to include data from environmental pollutants, including phthalates, in turn known to have the ability to disrupt lipid metabolism, glucose regulation, as well as vascular homeostasis [163]. Therefore, AI in general and ML in particular can leverage the associations between phthalate exposure and atherosclerosis and its features to develop mechanistic and predictive models integrating exposure biomarkers with early vascular changes.

### 5.4. Tips for Future Development

The integration of phthalate exposure data with cardiovascular and clinical features through AI represents a promising avenue for improving individualized risk prediction, identifying vulnerable or biologically fragile subgroups, and enhancing preventive cardiology through algorithms that explicitly incorporate environmental determinants. Such approaches may also inform public-health interventions targeting environmental cardiotoxins.

Future priorities in this field are likely to include the development of multimodal AI models and architecture capable of embedding chemical-exposure biomarkers and related information, cardiometabolic indicators, clinical variables, multi-omics data, behavioral patterns, and environmental features, to ultimately advance the understanding of environmental contributions to cardiovascular conditions, and IHD in particular, fostering precision medicine and preventative strategies.

## 6. Conclusions

Phthalate pollution is recognized as a global concern for both ecosystems and human health due to the extensive use of these compounds and their widespread distribution across all environmental matrices, despite progressive regulatory restrictions in major world regions. Although phthalates are primarily known as endocrine disruptors, they have more recently emerged as potential contributors to the pathogenesis of CVD and particularly IHD, the leading cause of mortality worldwide. Despite the limited number of published studies, all of which employ cross-sectional designs and which therefore cannot establish causality, exposure to selected phthalates appears to be significantly associated with key markers of atherosclerosis, the pathological hallmark of IHD. These include the number of atherosclerotic plaques, carotid IMT, and the echogenicity of both the intima–media complex and plaque structures, with associations persisting independently of traditional cardiovascular risk factors. Phthalates may also promote atherosclerosis by inducing apoptosis of endothelial and platelet cells, activating monocytes, macrophages, and neutrophils, thereby amplifying inflammatory responses, and increasing circulating thrombosis markers. Taken together, these findings suggest a potential role of phthalates in promoting atherosclerosis and, consequently, in the development of IHD, reinforcing the need to reduce phthalate use for cardiovascular prevention and to adequately monitor human exposure. Longitudinal, large-scale, multicenter studies with repeated exposure assessment are urgently needed to clarify the long-term cardiovascular effects of phthalate exposure. Moreover, the significant positive association between urinary phthalate levels and hs-cTn, a sensitive biomarker of myocardial injury, with effect sizes varying by age, sex, phthalate molecular weight, and with BMI and glycemic status acting as mediators, highlights the diverse cardiotoxic pathways through which phthalates may act and underscores the need for further investigation.

An increasing number of studies across cellular, animal, and human research show that exposure to phthalates and their metabolites causes significant changes at the cellular and molecular levels. These include oxidative stress and inflammation, mitochondrial dysfunction, epigenetic modifications, and lipid accumulation. In this context, induced pluripotent stem cells (iPSCs) represent a promising and innovative platform for studying how environmental toxins like phthalates impact the development of IHD. Using human blood-derived iPSC lines, researchers can generate both 2D and 3D heart tissue models that closely replicate native structure and function, facilitating detailed studies of gene-environment interactions and individual genetic susceptibility to phthalates. Such research has the potential to identify harmful effects and uncover disease mechanisms early on—possibly before these effects are detectable in large population studies—thereby advancing our understanding and prevention of IHD related to environmental exposures [164].

Finally, future studies might take advantage of the technological developments in terms of novel instruments, consumer tools and, ultimately, AI models and approaches, enabling the discovery of mechanistic interplays between environmental determinants, their biological and clinical effects, and ultimately fostering the optimization of the clinical outcome.

## Data Availability

No new data was created.

## Abbreviations

The following abbreviations are used in this manuscript:

5mdC	5-methyl-2-deoxycytidine
AI	Artificial intelligence
AMI	Acute myocardial infarction
ASCVD	Atherosclerotic cardiovascular diseases
AUC	Area under the curve
AUROC	Area under the receiving operating characteristics curve
BBP	Butyl benzyl phthalate
BBzP	Butylbenzyl phthalate
BMI	Body mass index
bw	Body weight
CAC	Coronary artery calcium
CCTA	Coronary computed tomography angiography
cGAN	Conventional generative adversarial network
CHD	Coronary heart disease
CNN	Convolutional neural network
CT	Computed tomography
CVD	Cardiovascular disease
dG	2-deoxyguanine
DBP	Di-n-butyl phthalate
DEHP	2-ethylhexyl phthalate
DEP	Diethyl phthalate
DIBP	Diisobutyl phthalate
DIDP	Di-iso-decyl phthalate
DINP	Diisononyl phthalate
DIPP	Diisobutyl phthalate
DL	Deep Learning
DMP	Dimethyl phthalate
DnBP	Di-n-butyl phthalate
DnOP	di-n-octyl phthalate
DOP	Dinoctyl phthalate
eGFR	Estimated glomerular filtration rate
EBP	Elevated blood pressure
EHR	Electronic health record
EMP	Endothelial microparticle
FPG	Fasting plasma glucose
GBM	Gradient boosting machine
GSH	Glutathione
GSM	Gray-scale median
HMW	High molecular weight
hs-CRP	High-sensitivity C-reactive protein
hscTn	High-sensitivity cardiac troponin I
IHD	Ischemic heart disease
IMT	Intima–media thickness
IL	Interleukin
lncRNA	Long non-coding RNA
LLE-GC-MS	Liquid–liquid extraction/gas chromatography/mass spectrometry
LMW	Low molecular weight
MBP	Mono-butyl phthalate
MBzP	Monobenzyl phthalate
MCPP	Mono-3-carboxy propyl phthalate
MDA	Malondialdehyde
MECPP	mono-2-ethyl-5-carboxypentyl phthalate
MEHHP	Mono(ethyl-5-hydroxyhexyl) phthalate
MEHOP	Mono(2-ethly-5-oxoheyl) phthalate
MEHP	Mono (2 ethylhexyl) phthalate
MEP	Mono-ethyl phthalate
MiBP	Mono-isobutyl phthalate
miRNA	Micro RNA
ML	Machine learning
MMP	Mono-methyl phthalate
MnBP	Mono-n-butyl phthalate
mtDNA	Mitochondrial DNA
PLBoost	Phthalate boosting
PMP	Platelet microparticle
ROS	Reactive oxygen species
SDI	Sociodemographic index
SMC	Smooth muscle cell
SOD	Superoxide dismutase
T2D	Type 2 diabetes
TDI	Tolerable daily intake
VSMC	Vascular smooth muscle cell
